# Kapitza Length at Solid–Liquid Interface: From Nanoscale to Microscale

**DOI:** 10.1002/smsc.202400626

**Published:** 2025-03-08

**Authors:** Wentao Chen, Gyoko Nagayama

**Affiliations:** ^1^ Department of Mechanical Engineering Kyushu Institute of Technology Kitakyushu Fukuoka 804‐8550 Japan

**Keywords:** Kapitza length, molecular dynamics simulations, scale effects, solid–liquid interfaces, thermal energy transports

## Abstract

Understanding thermal energy transport at solid–liquid interfaces is critical for enhancing the performance of nano‐ or microscale systems. Although extensive studies have addressed the interfacial thermal resistance, known as Kapitza length, its impact on interfacial heat transfer from nanoscale to microscale remains limited. This study explores the Kapitza length at hydrophilic and hydrophobic solid–liquid interfaces under constant heat flux or overall temperature difference using nonequilibrium molecular dynamics simulations. The findings reveal that Kapitza length remains nearly constant under constant heat flux, while it is comparable to the liquid film thickness under constant overall temperature differences in both nano‐ and microscale systems. Notably, a giant Kapitza length of 1382 nm was found at a hydrophobic solid–liquid interface with a 1082 nm‐thick liquid film. Upon comparing Kapitza length obtained from simulation with experimental results, three primary regimes of solid–liquid interfacial heat transfer are identified: phononic, transition, and conductive regimes. These insights highlight the substantial effect of Kapitza length on solid–liquid interfacial heat transfer from nano‐ to microscales, offering potential avenues for advanced thermal management in nano‐ or microscale systems.

## Graphical abstract

This study addresses the critical challenge of understanding solid–liquid interfacial heat transfer from nanoscale to microscale, focusing on how liquid film thickness affects Kapitza length under the constant overall temperature difference or heat flux. Key findings from this study reveal three primary regimes of interfacial heat transfer: phononic, transition, and conductive regimes.

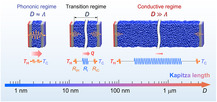

## 1 Introduction

**Figure 1:**
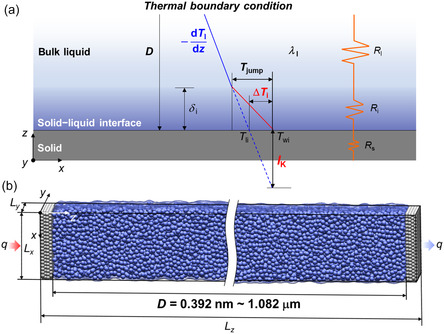
a) Schematic of the definition for Kapitza length at the solid–liquid interface. b) NEMD simulation system.

**Figure 2:**
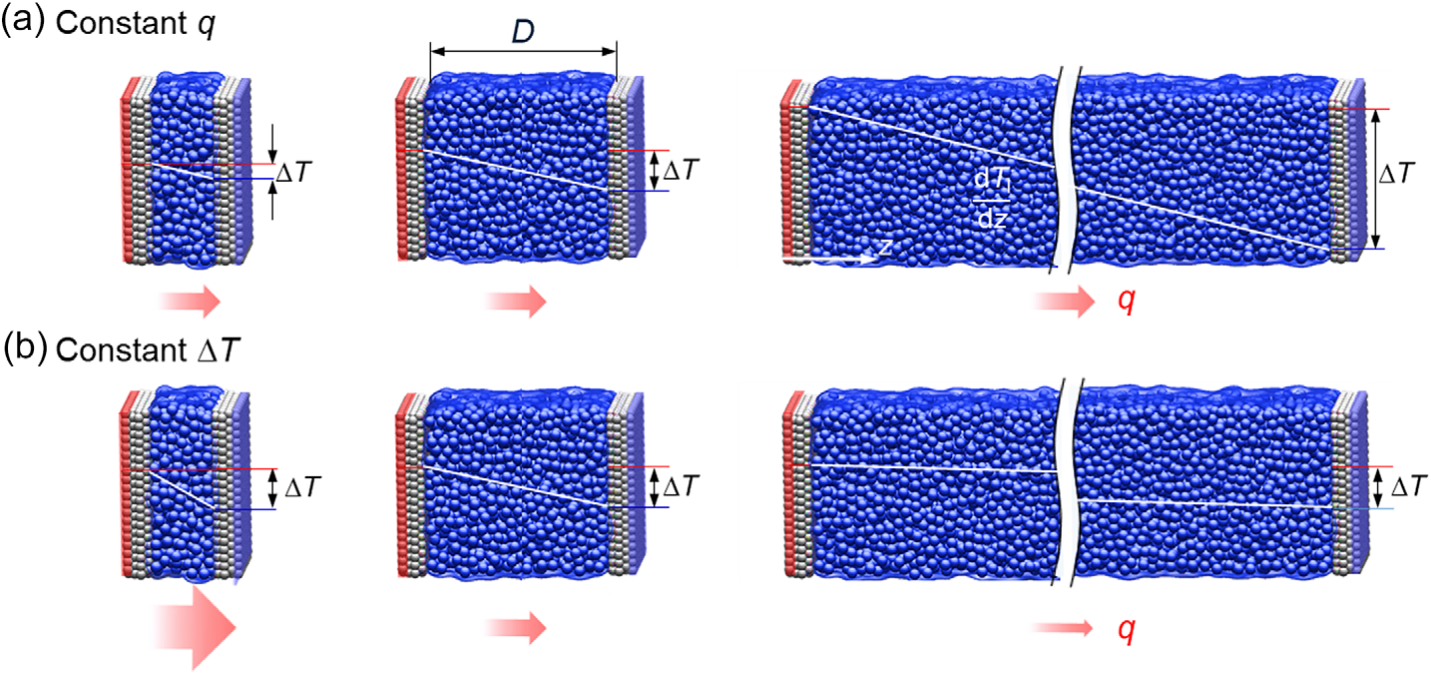
Schematics of heating methods for thermal energy transport across a solid–liquid interface with various liquid film thicknesses *D*: a) constant heat flux *q* and b) constant overall temperature difference Δ*T* between the heating and cooling thermostats.

Solid–liquid interfacial heat transfer is crucial not only in conventional heat exchangers but also in emerging technologies such as nanofluids,^[^ ^1^, ^2^ ^]^ biochips,^[^ ^3^ ^]^ fuel cells,^[^ ^4^, ^5^ ^]^ thermoelectrics,^[^ ^6^, ^7^ ^]^ and energy storage.^[^ ^8^, ^9^ ^]^ The miniaturization of nano/microscale electronic devices has intensified the focus on thermal transport at the solid–liquid interface.^[^ ^10^, ^11^ ^]^ Notably, noncontinuous boundary conditions play a significant role as the characteristic lengths of the system reach nano/microscale dimensions.^[^ ^12^, ^13^, ^14^, ^15^ ^]^ **Figure** **1**a depicts a temperature jump *T*_jump_ at an interface owing to a mismatch between the solid and liquid, corresponding to the interfacial thermal resistance *R*_i_. *T*_jump_ was initially determined by P. L. Kapitza at the copper–helium interface^[^ ^16^ ^]^ in 1941. The equivalent thickness of *R*_i_ due to *T*_jump_ is thermal slip length, which is generally defined as Kapitza length *l*_K_ (= Δ*T*_i_/(−d*T*_l_/d*z*) = *λ*_l_ *R*_i_ = *λ*_l_ Δ*T*_i_/*q*, where *λ*_l_ is the thermal conductivity of the liquid, Δ*T*_i_ is the interfacial temperature difference between the solid and liquid, *q* is the heat flux, and d*T*_l_/d*z* is the temperature gradient in a bulk liquid). There are two heating methods for studying *l*_K_ as shown in **Figure** **2**: a) constant *q* and b) constant overall temperature difference Δ*T* between the heating and cooling thermostats. The constant *q* is typically utilized in experimental setups, whereas the constant Δ*T* is commonly employed in simulations.

Extensive studies have examined the factors that influence thermal transport at solid–liquid interfaces, for example, surface wettability,^[^ ^17^, ^18^, ^19^ ^]^ self‐assembled monolayers,^[^ ^20^, ^21^, ^22^ ^]^ temperature,^[^ ^23^, ^24^, ^25^ ^]^ nanostructures,^[^ ^26^, ^27^, ^28^, ^29^ ^]^ nanoparticles,^[^ ^30^, ^31^ ^]^ liquid pressure,^[^ ^32^, ^33^ ^]^ and liquid film thickness.^[^ ^34^, ^35^, ^36^ ^]^ Owing to the complexity of the experiments, only a limited number of studies have addressed thermal energy transport at nano/microscale solid–liquid interfaces. To date, the transient techniques such as time‐domain thermoreflectance (TDTR),^[^ ^37^, ^38^, ^39^ ^]^ the 3ω method,^[^ ^40^ ^]^ and the steady‐state method according to the American Society for Testing and Materials (ASTM) D5470 standard^[^ ^41^, ^42^ ^]^ have been utilized to measure interfacial thermal conductance *G* or *R*_i_ at the solid–liquid interface. The correlation between *G* and surface wettability has been specifically investigated using the TDTR method. It was reported that increased hydrophobicity led to lower *G* at the aluminum (Al)–water (H_2_O) interface,^[^ ^37^ ^]^ consistent with observations at the gold (Au)–H_2_O interface.^[^ ^38^, ^39^ ^]^ In these cases, the Al surface investigated by Ge et al.^[^ ^37^ ^]^ and the Au surface investigated by Harikrishna et al.^[^ ^38^ ^]^ were chemically functionalized with a self‐assembled monolayer. Tomko et al. examined the unmodified Au surface and found that the solid–liquid interface notably influenced the acoustic phonon propagation.^[^ ^39^ ^]^ Utilizing a bidirectional and differential 3ω method, *R*_i_ was measured at the glass–H_2_O interface with a 0.2 μm‐thick intervening air layer.^[^ ^40^ ^]^ However, both the TDTR and 3ω methods ignore the liquid film thickness *D* in predicting the experimental data of *R*_i_. On the other hand, the steady‐state method, which adheres to the ASTM D5470 standard, was applied to assess *R*_i_ at the Al–silicone oil^[^ ^41^ ^]^ and silicon (Si)–H_2_O^[^ ^42^ ^]^ interfaces.

**Table** **1** summarizes the estimated *l*_K_ values using *R*_i_ or *G* data obtained in the aforementioned works. *l*_K_ ranges from 3 to 10 nm using the TDTR method, while it is ≈4.511 μm by the 3ω method. For the steady‐state method, *l*_K_ is 4.225 μm at the hydrophilic Si–H_2_O interface when *D* is 500, and 90.225 μm at the Al–silicone oil interface when *D* is 46.7 μm. The estimated *l*_K_ obtained using the steady‐state method is much higher than the values obtained using transient methods. Additionally, the values of *l*_K_ estimated using the Nusselt number in a developed region of parallel‐plate microchannels were 150 and 250 μm at the hydrophilic and hydrophobic interfaces, respectively.^[^ ^15^, ^43^ ^]^ The discrepancy in *l*_K_ among the different experimental methods is remarkable, which might be attributed to the limitations of the experimental setup. Although constant *q* is typically utilized in experiments, the transient method usually lacks detailed information on *q*, *D*, and Δ*T*. In comparison, large Δ*T* and *q* for small *D* are challenging in measurements using the steady‐state method. Consequently, Δ*T* and *q* in different experimental setups are not comparable.

**Table 1:** Experimental and NEMD studies on thermal transport at solid–liquid interfaces.

Authors	Materials	Liquid film thickness *D* [nm]	Kapitza length *l*_K_ [nm]	
Experiments	–	–	Hydrophilic	Hydrophobic
Harikrishna et al. (TDTR)^[^ ^38^ ^]^	Au–H_2_O	–	3.16a)	9.23a)
Tomko et al. (TDTR)^[^ ^39^ ^]^	Au–H_2_O	–	8.82a)	–
Ge et al. (TDTR)^[^ ^37^ ^]^	Al–H_2_O	–	3.00	10.00
Jiao et al. (3ω)^[^ ^40^ ^]^	Glass–H_2_O	–	–	4511a)
Yuan et al. (ASTM D5470)^[^ ^41^ ^]^	Al–silicone oil	46 700	–	90 225a)
Yu et al. (ASTM D5470)^[^ ^42^ ^]^	Si–H_2_O	500 000	4,225a)	–
Mitoma et al. (estimated)^[^ ^43^ ^]^	Si–H_2_O	100 000	150 000	250 000
Nagayama et al. (estimated)^[^ ^15^ ^]^	Si–H_2_O	100 000	150 000	–
NEMD simulations	–	–	Hydrophilic	Hydrophobic
Zhang et al. (Δ*T* = 33 K)^[^ ^49^ ^]^	Pt–Ar	1.66–35.33	11.75–17.46	–
Liu et al. (Δ*T* = 30 K)^[^ ^35^ ^]^	Pt–Ar	0.25–5.81	0.12–3.77a)	–
Alosious et al. (Δ*T* = 100 K)^[^ ^46^ ^]^	Graphene–H_2_O	2.00–8.00	–	11.07–11.54
Alexeev et al. (Δ*T* = 100 K)^[^ ^45^ ^]^	Graphene–H_2_O	1.00–5.00	–	28.00–29.30a)
Gonçalves et al. (Δ*T* = 60 K)^[^ ^48^ ^]^	Si–H_2_O	3.70–16.30	7.79–10.44	–
Masuduzzaman et al. (Δ*T* = 50 K)^[^ ^47^ ^]^	Ag–Ar	3.27–7.35	0.50–0.72	–
Wang et al. (Δ*T* = 60 K)^[^ ^44^ ^]^	Ag–Ar	0.36–1.62	0.41–2.44a)	2.31–10.00a)

a) Based on an analysis by the present authors.

Owing to experimental limitations, nonequilibrium molecular dynamics simulation (NEMD) has been employed as an expedient method to investigate *l*_K_ in nanoscale systems. The constant *q* utilized in experiments is rarely employed in NEMD simulations. Alternatively, the existing simulations are usually performed under constant Δ*T*.^[^ ^35^, ^44^, ^45^, ^46^, ^47^, ^48^, ^49^ ^]^ At the platinum (Pt)–argon (Ar) interface, the estimated *l*_K_ decreased significantly with reduced *D* spanning 0.25–5.81 nm.^[^ ^35^ ^]^ Similar outcomes for *l*_K_ were observed at the silver (Ag)–Ar interface with *D* ranging from 0.36 to 1.62 nm.^[^ ^44^ ^]^ In contrast, *l*_K_ remained constant at the graphene–H_2_O interface with *D* in the range of 1–5 nm^[^ ^45^ ^]^ and 2–8 nm^[^ ^46^ ^]^ and at the Ag–Ar interface with *D* in the range of 3.27–7.35 nm.^[^ ^47^ ^]^ At the Si–H_2_O interface, *l*_K_ exhibited nonmonotonic variations with *D* ranging from 3.7 to 16.3 nm.^[^ ^48^ ^]^ Furthermore, *l*_K_ at the Pt–Ar interface monotonically increased with increasing *D* when *D* < 7.85 nm but was consistent in the range 7.85 < *D* < 35.33 nm.^[^ ^49^ ^]^

The aforementioned NEMD simulations were conducted under significant Δ*T* which exceeded 30 K (up to 100 K) for liquid films with *D* varying from 0.25 to 35.33 nm. Although a large Δ*T* is often used for nanoscale liquid films for computational expediency, it is less representative of practical conditions owing to the considerable *q* and d*T*_l_/dz. For instance, *q* and average d*T*_l_/dz values are ≈509 MW m^−2^ and 9.3 × 10^8^ K m^−1^, respectively, for a water film at *D* = 35.33 nm and Δ*T* = 30 K. Such a high heat flux which exceeds the critical heat flux for nucleate boiling is impractical for experimental studies on solid–liquid interfacial heat transfer. Therefore, a smaller Δ*T* employed in a larger *D* simulation system is desirable in NEMD. However, performing NEMD simulations with *D* > 100 nm for smaller Δ*T* or constant *q* is still challenging owing to the high computational cost required to achieve accurate results.

This critical gap in the current understanding of *l*_K_ at the nano/microscale is addressed in this study. We conducted comprehensive NEMD simulations to explore the effects of *D*, Δ*T*, and *q* on *l*_K_ at both Pt–Ar and Si–H_2_O interfaces. A challenge in NEMD simulations is to examine *l*_K_ for *D* exceeding 100 nm under constant Δ*T* or constant *q*. To improve the accuracy of the simulation results, the steady state was reached using a time window of 500 ns microcanonical ensemble (NVE). The density and temperature profiles, interfacial temperature differences, and temperature gradients were obtained based on 25 ns data sampling. A maximum *D* of 1082 nm and a minimum Δ*T* of 4 K resulted in a *q* of 0.1 MW m^−2^ and d*T*_l_/d*z* of 10^6^ K m^−1^. The vibrational density of states (VDOSs) of the interfacial solid and liquid layers and those overlaps were analyzed. Furthermore, we calculated *R*_i_, *G*, and *l*_K_ and compared the results with the published data for a wide range of *D* from the nanoscale to the microscale. The results of this study not only deepen our fundamental understanding of solid–liquid interfacial heat transfer but also provide practical insights for the design and optimization of nano‐ or microscale systems.

## 2 Results and Discussion

### 2.1 Effects of Heat Flux and Overall Temperature Difference on Kapitza Length

**Figure 3:**
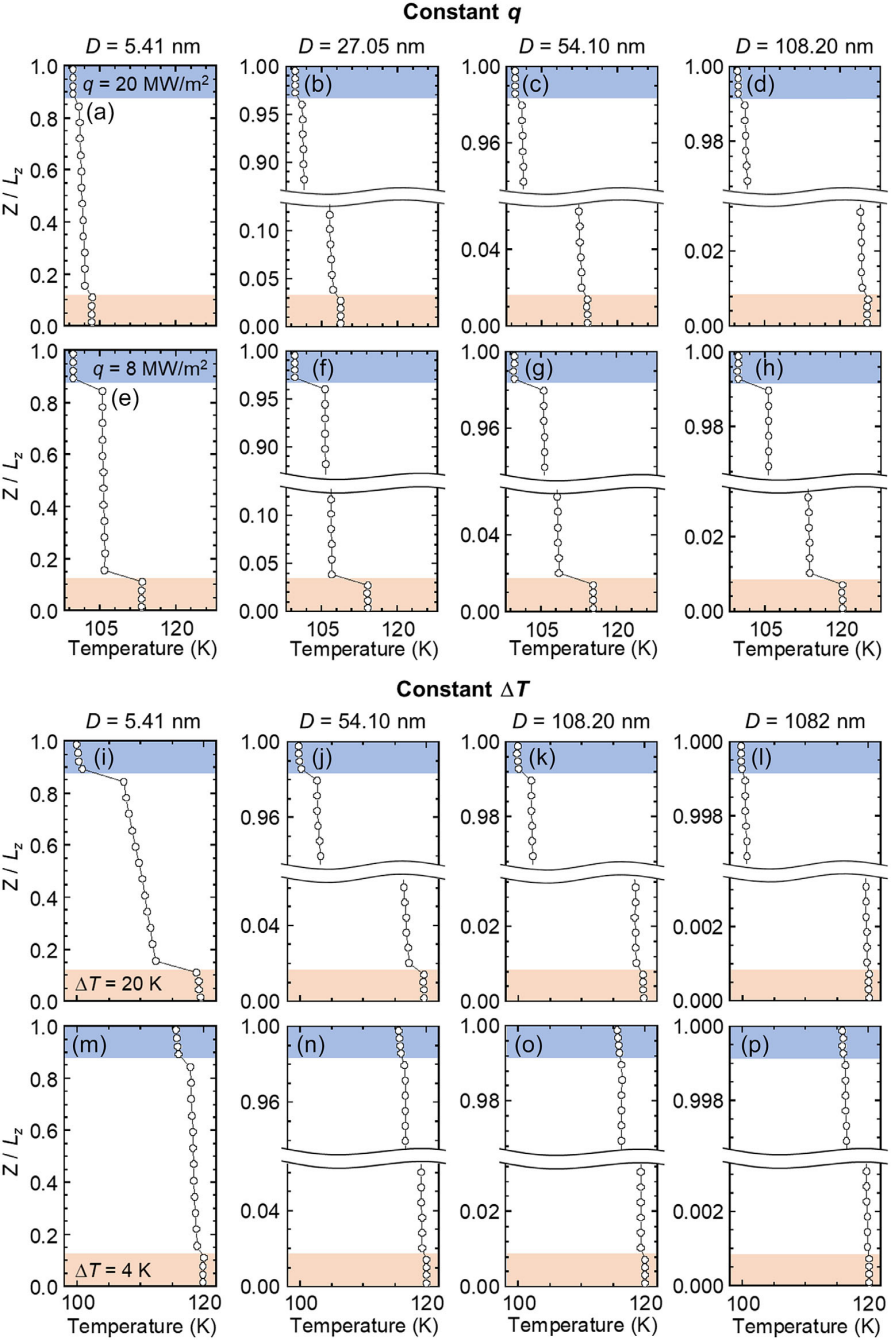
Temperature profiles under constant heat flux a–d) *q* = 20 MW m^−2^ and e–h) *q* = 8 MW m^−2^ as liquid film thickness *D* varies from 5.41 to 108.2 nm and under the constant overall temperature difference i–l) Δ*T* = 20 K and m–p) Δ*T* = 4 K as liquid film thickness *D* varies from 5.41 to 1082 nm in hydrophilic Pt–Ar systems.

**Figure** **3** illustrates the temperature distributions calculated for 25 ns data sampling procedures in the steady state under both constant *q* and Δ*T*. The standard error (SE) for the temperature was less than 0.062 K, and the relative standard error (RSE) was below 0.052% (details provided in Table S1 and S2, Supporting Information), confirming the reliability of the temperature data presented in Figure 3. The interfacial temperatures of the solid and liquid, denoted as *T*_wi_ and *T*_li_, respectively, obtained from the temperature extrapolation of the linear fit for temperature distribution, were used to calculate Δ*T*_i_ (= *T*_wi_ − *T*_li_). The SE and RSE of Δ*T*_i_ were less than 0.01 K and 1.7%, respectively (refer to Section S3, Supporting Information).

Under constant *q* of 8 or 20 MW m^−2^, Δ*T*_i_ and d*T*_l_/d*z* were consistent as *D* increased from 5.41 to 108.2 nm, as illustrated in Figure 3a–h. However, Δ*T*_i_ and d*T*_l_/d*z* decreased with increasing *D* under constant Δ*T* of 4 or 20 K for hydrophilic systems, as shown in Figure 3i–p. Figure S3, Supporting Information, presents similar results for the hydrophobic systems, where Δ*T*_i_ is larger and d*T*_l_/d*z* is smaller compared with hydrophilic cases. Comprehensive results on Δ*T*_i_ and d*T*_l_/d*z* for all scenarios are compiled in Figure S5 and Table S7 and S8, Supporting Information. Notably, at Δ*T* = 4 K, Δ*T*_i_ is nearly stable, whereas d*T*_l_/d*z* decreases sharply when *D* exceeds 54.1 nm. Additionally, the values of d*T*_l_/d*z* are exceedingly small at Δ*T* = 4 K and *D* = 1082 nm, being 0.0026 and 0.001 for hydrophilic and hydrophobic cases, respectively. These results suggest that an exceedingly small d*T*_l_/d*z* can result in a significant *l*_K_ at the solid–liquid interface.

**Figure 4:**
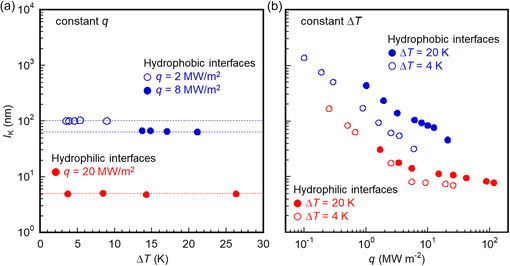
a) Kapitza length *l*_K_ versus overall temperature difference Δ*T* under heat flux *q* = 20 MW m^−2^ in hydrophilic cases and *q* = 2 and 8 MW m^−2^ in hydrophobic cases. b) Kapitza length *l*_K_ versus heat flux *q* under overall temperature difference Δ*T* = 4 and 20 K in hydrophilic and hydrophobic cases. The dotted lines serve as a guide for the eye.

**Figure 5:**
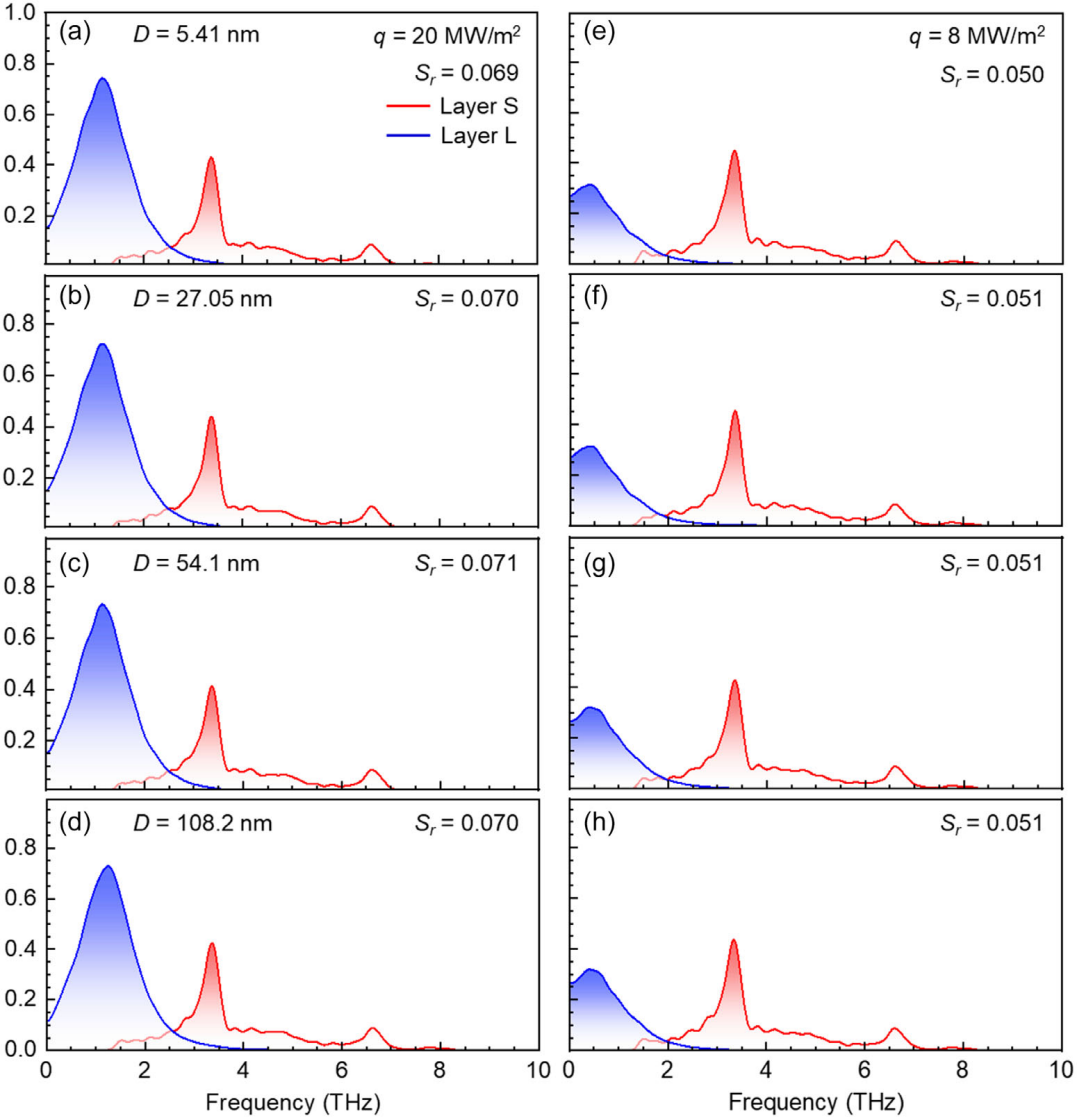
VDOSs of layers S and L under the constant heat flux a–d) *q* = 20 MW m^−2^ and e–h) *q* = 8 MW m^−2^ at *D* = 5.41–108.2 nm. Layer S and layer L are the cooling interfacial solid and liquid layers, respectively, in contact with each other.

**Table** **2** shows that Δ*T* increases with increasing *D* under the constant *q*, whereas *q* decreases with increasing *D* under the constant Δ*T*, as demonstrated in Table S7 and S8, Supporting Information,. Moreover, **Figure** **4** illustrates *l*_K_ for two scenarios: under constant *q* of 2, 8, or 20 MW m^−2^ for *D* = 5.41–324.6 nm and under constant Δ*T* of 4 or 20 K for *D* = 0.392–1082 nm. When *D* = 324.6 nm, *l*_K_ is obtained only under *q* of 2 MW m^−2^ because the temperature of the heating wall exceeds the critical temperature of Ar (150.9 K) under constant *q* of 8 and 20 MW m^−2^. Figure 4a shows that *l*_K_ remains nearly stable under a constant *q* for various Δ*T*, owing to the similar phonon–phonon coupling between the interfacial solid and liquid layers. This stability is supported by **Figure** **5**, which illustrates that the overlap of the VDOSs *Sr* between the interfacial solid layer *S* and the liquid layer *L* remains consistent under the constant *q* of 8 and 20 MW m^−2^. On the other hand, under the constant Δ*T* of 4 or 20 K, *l*_K_ increases sharply with decreasing *q* as illustrated in Figure 4b. This suggests that *q* is a critical factor in controlling *l*_K_ ($=\lambda_{l}\Delta T_{i}/q$) at the solid–liquid interface. Therefore, the effect of *D* on *l*_K_ under a constant Δ*T* will be further examined in subsequent sections.

**Table 2:** Simulation results for the Pt–Ar interface under constant heat flux.

*D* [nm]	*q* [MW m^−2^]	Δ*T* [K]	Δ*T*_i_ [K]	−d*T*_l_ */*d*z* [K nm^−1^]	*l*_K_ [nm]	*G* [MW m^−2^ K^−1^]
Hydrophilic interfaces						
5.41	20	3.7	1.19	0.231	5.15	16.81
27.05	20	8.4	1.14	0.227	5.02	17.54
54.10	20	14.3	1.17	0.224	5.22	17.09
108.20	20	26.3	1.15	0.223	5.16	17.39
Hydrophobic interfaces						
5.41	8	13.7	6.19	0.096	64.48	1.29
27.05	8	14.8	6.15	0.092	66.85	1.30
54.10	8	17.0	6.14	0.092	66.74	1.30
108.20	8	21.1	6.02	0.090	66.89	1.33
5.41	2	3.5	1.70	0.017	100.24	1.17
27.05	2	3.9	1.72	0.017	101.18	1.16
54.10	2	4.6	1.81	0.018	100.02	1.10
108.20	2	5.4	1.77	0.017	102.88	1.13
324.60	2	9.0	1.72	0.017	100.86	1.16

### 2.2 Theoretical Analysis Under Constant Overall Temperature Difference

The Kapitza length *l*_K_ can be estimated by extrapolating the temperature profile from the bulk liquid to the solid, analogous to the velocity slip length. Within the context of an NEMD system, as depicted in Figure 1b, *l*_K_ can be computed using the following equation

(1) $$l_{K}=\frac{\Delta T_{i}}{\left(-\frac{dT_{l}}{dz}\right)}=\frac{\Delta T_{i}}{\left(\frac{\Delta T-2T_{\text{jump}}}{D-2\delta_{i}}\right)}$$

where *δ*_i_ is the thickness of the solid–liquid interfacial region. By applying the triangle proportionality theorem $\left(l_{K}+\delta_{i}\right)/l_{K}=T_{\text{jump}}/\Delta T_{i}$ to Equation (1), the ratio Δ*T*_i_/Δ*T* can be calculated as

(2) $$\frac{\Delta T_{i}}{\Delta T}=\frac{1}{D/l_{K}+2}=\frac{1}{D/(R_{i}\lambda_{l})+2}=\frac{1}{DG/\lambda_{l}+2}$$

where $R_{i}=\Delta T_{i}/q=l_{K}/\lambda_{l}$. The interfacial thermal conductance *G* can be determined using the reciprocal of the interfacial thermal resistance

(3) $$G=\frac{1}{R_{i}}=\frac{q}{\Delta T_{i}}=\frac{\lambda_{l}}{\Delta T_{i}}\left(-\frac{dT_{l}}{dz}\right)$$

**Figure 6:**
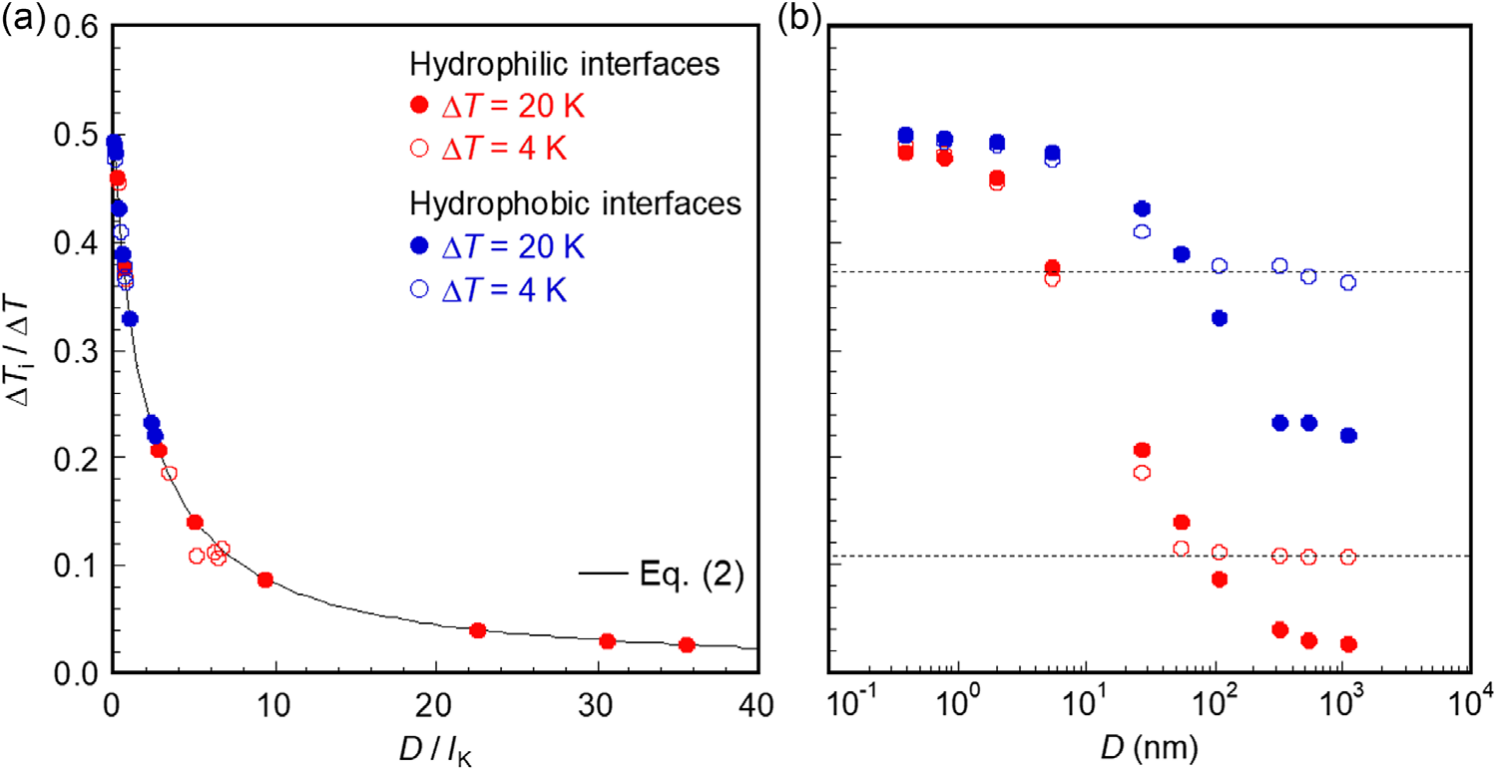
The ratio of the temperature difference at the solid–liquid interface Δ*T*_i_ to the overall temperature difference Δ*T*, represented as Δ*T*_i_/Δ*T*, plotted as a function of a) the ratio *D*/*l*_K_, and b) the liquid film thickness *D*. The dotted lines serve as a guide for the eye.

In scenarios with constant *q* and Δ*T*_i_, *l*_K_ remains relatively unchanged, as indicated in Table 2. However, in cases with a similar Δ*T*_i_, a smaller *q* results in an increase in *l*_K_ with increasing *D*. To examine the influence of *D* on *l*_K_, we maintained a constant Δ*T* of either 4 or 20 K in the systems. The Δ*T*_i_/Δ*T* results for all simulation cases are presented in **Figure** **6** as a function of *D*/*l*_K_ or *D*. In Figure 6a, Δ*T*_i_/Δ*T* increases as *D*/*l*_K_ decreases, reaching ≈0.5 as *D*/*l*_K_ approaches zero. The simulation data for Δ*T*_i_/Δ*T* (represented by dots and circles) closely align with the theoretical predictions (illustrated by a solid line) derived from Equation (2), which is indicative of a predominantly linear temperature gradient within the bulk liquid. In Figure 6b, Δ*T*_i_/Δ*T* is higher in hydrophobic cases compared with hydrophilic scenarios. When a liquid monolayer is confined between two solid walls, Δ*T*_i_/Δ*T* is ≈0.5. At *D* greater than 54.1 nm, Δ*T*_i_/Δ*T* nearly stabilizes at Δ*T* = 4 K. Consequently, *l*_K_ can be predicted using Equation (2), assuming a constant Δ*T*_i_/Δ*T* in larger‐scale simulation systems.

### 2.3 Heat Flux and Kapitza Length Under Constant Overall Temperature Difference

**Figure 7:**
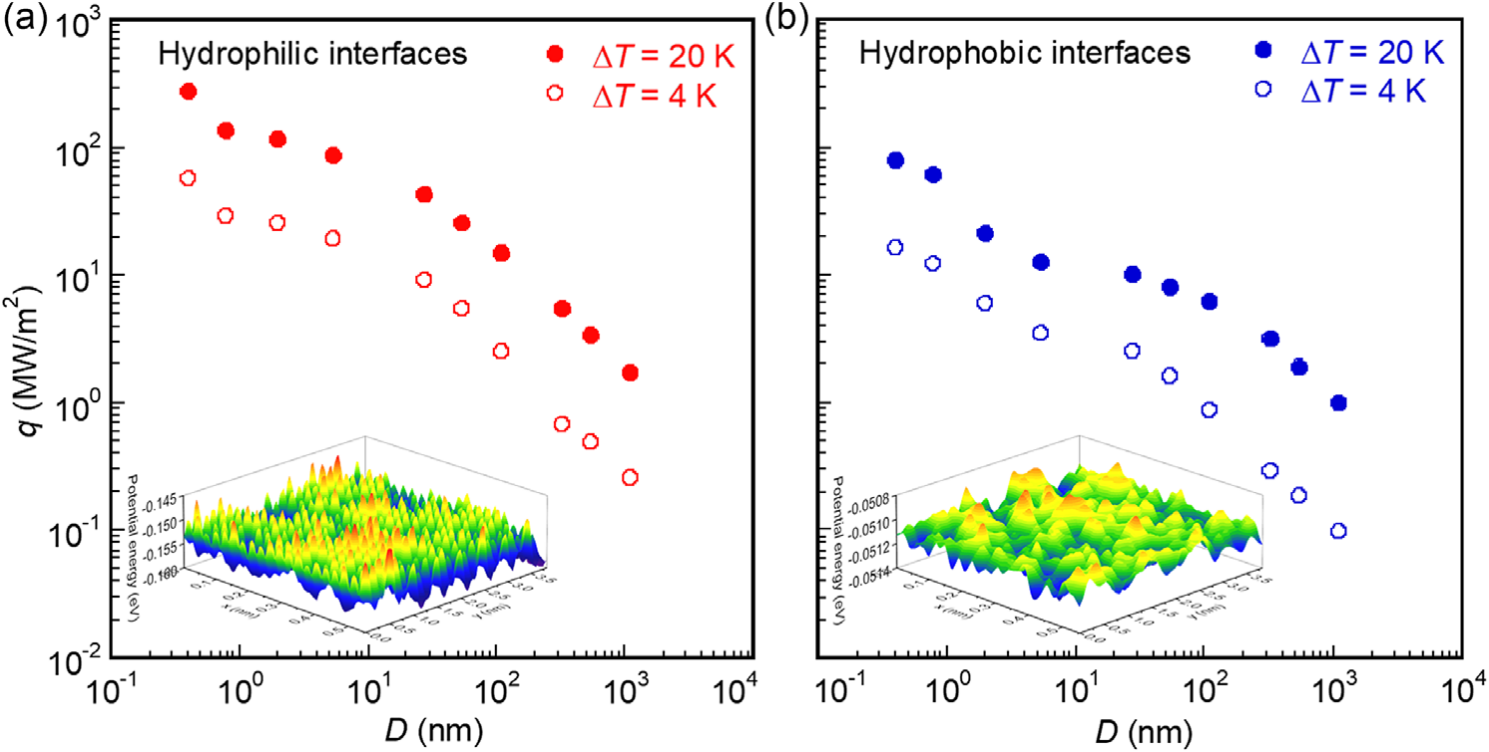
Heat flux *q* versus liquid film thickness *D* of 0.392 nm–1.082 μm under an overall temperature difference of Δ*T* = 20 and 4 K in a) hydrophilic and b) hydrophobic cases. The insets show the potential energy of the first liquid layer adjacent to the cooling solid surfaces in the *xy* plane at Δ*T* = 20 K and *D* = 5.41 nm.

To explore the relationship between solid–liquid interfacial heat transfer and the liquid film thickness *D*, we calculated *q*, *R*_i_, and *l*_K_ at the solid–liquid interface under Δ*T* = 4 K and 20 K. The heat flux *q* decreased by two to three orders of magnitude as *D* increased from 0.392 to 1082 nm, with higher values observed in the hydrophilic cases compared with the hydrophobic cases, as depicted in **Figure** **7**. The insets in Figure 7 show the potential energy of the first interfacial liquid layer adjacent to the cooling solid surfaces in the *xy* plane at Δ*T* = 20 K and *D* = 5.41 nm for both hydrophilic and hydrophobic cases. The hydrophilic case exhibits a lower average potential energy (−0.154 eV) due to the stronger solid–liquid interaction, compared with hydrophobic case (−0.051 eV). Additionally, Figure S1 and S2, Supporting Information, demonstrate that interfacial liquid layers in hydrophilic cases distribute more orderly with higher density than in hydrophobic cases, consistent with the finding of our previous study.^[^ ^19^ ^]^

**Figure 8:**
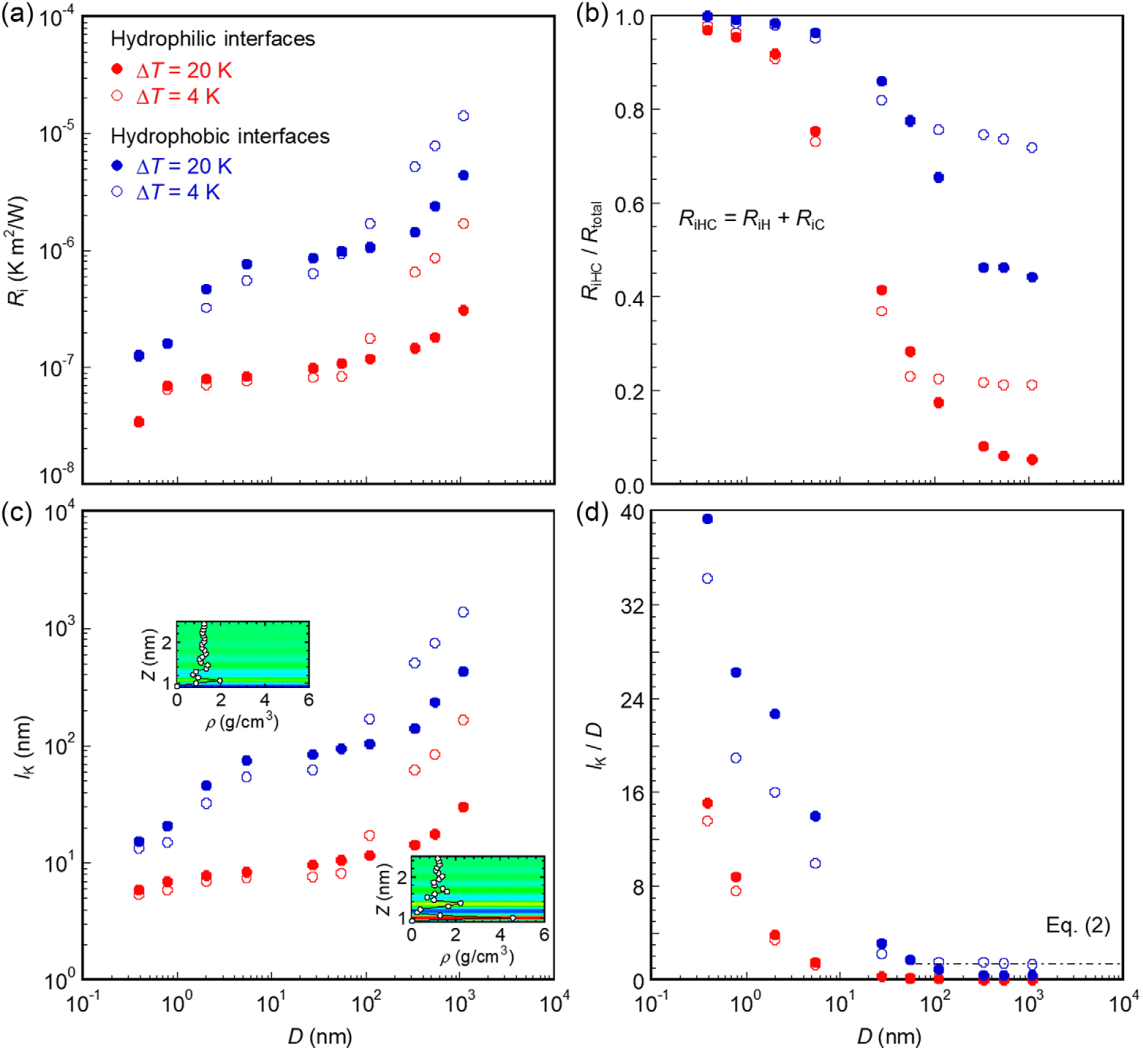
a) Interfacial thermal resistance *R*_i_, b) the ratio *R*_iHC_/*R*_total_, c) Kapitza length *l*_K_, and d) the ratio *l*_K_/*D* versus liquid film thickness *D* under an overall temperature difference of Δ*T* = 20 and 4 K in hydrophilic and hydrophobic cases. The insets in (c) show the density distributions of the liquid interfacial region in the *xz* plane for the hydrophilic and hydrophobic cases at Δ*T* = 20 K and *D* = 5.41 nm.

Correspondingly, **Figure** **8a** **,**c depicts an increase in *R*_i_ and *l*_K_ with increasing *D*. The larger values were observed in the hydrophobic case compared to the hydrophilic case. The density of solid‐like liquid layers in the interfacial region of the hydrophilic case is much higher than that of the hydrophobic case, as illustrated in the inserted density distributions in Figure 8c. Moreover, the thermal conductivity $\lambda_{i}$ of interfacial liquid layers with a thickness of 1.38 nm adjacent to the cooling wall in hydrophilic case exceeds that in hydrophobic case at Δ*T* = 20 K and *D* = 5.41 nm, as shown in Figure S10, Supporting Information. The higher density of interfacial liquid layers indicates that more liquid atoms participate in the interfacial heat transfer, resulting in lower interfacial thermal resistance. This finding aligns with heat transfer across the graphene–H_2_O interface.^[^ ^50^ ^]^ Notably, *R*_i_ and *l*_K_ increase more sharply under Δ*T* = 4 K for *D* ranging from 54.1 to 1082 nm due to the extremely small d*T*_l_/d*z*. At the hydrophobic solid–liquid interface, *l*_K_ is 1382 nm for the 1082 nm‐thick liquid film under Δ*T* = 4 K. This represents the first reported instance of a high *l*_K_ comparable to *D* and aligns with the experimental findings of single‐phase convective heat transfer characteristics in microchannel flows.^[^ ^15^ ^]^ Figure 8b,d shows a decline in *R*_iHC_/*R*_total_ and *l*_K_/*D* with increasing *D*, owing to the increased thermal resistance of bulk liquid *R*_b_. The heat flux *q* can be calculated by $q=\Delta T_{l}/R_{\text{total}}=\Delta T_{l}/(R_{\text{iHC}}+R_{b})$, where Δ*T*_l_ is the temperature difference of liquid. The reduction in the ratio *R*_iHC_/*R*_total_ indicates that the relative influence of *R*_iHC_ on *q* diminishes as *D* increases. Consequently, the effects of interfacial thermal resistance on solid–liquid interfacial heat transfer decrease with increasing *D*. At *D* ≥ 54.1 nm, *l*_K_/*D* nearly stabilizes at Δ*T* = 4 K and aligns closely with the theoretical predictions (highlighted by dashed and dash‐dotted lines) derived from Equation (2).

Under a constant Δ*T* of 4 or 20 K, increasing *D* results in a reduction in *q*, as depicted in Figure 7. This decrease in *q* can lead to an increase in *R*_i_, according to Equation (3). Thus, Figure 4b demonstrates that *l*_K_ decreases with increasing *q* when Δ*T* is held constant. However, as illustrated in Table 2 and Figure 4a, *l*_K_ remains consistent for constant *q* and Δ*T*_i_ at both hydrophilic and hydrophobic Pt–Ar interfaces, with *D* ranging from 5.41 to 324.6 nm. Consequently, the increase in *l*_K_ with a larger *D* is attributed to a smaller *q* under the constant Δ*T*.

### 2.4 Dependence of Kapitza Length on Liquid Film Thickness

**Figure 9:**
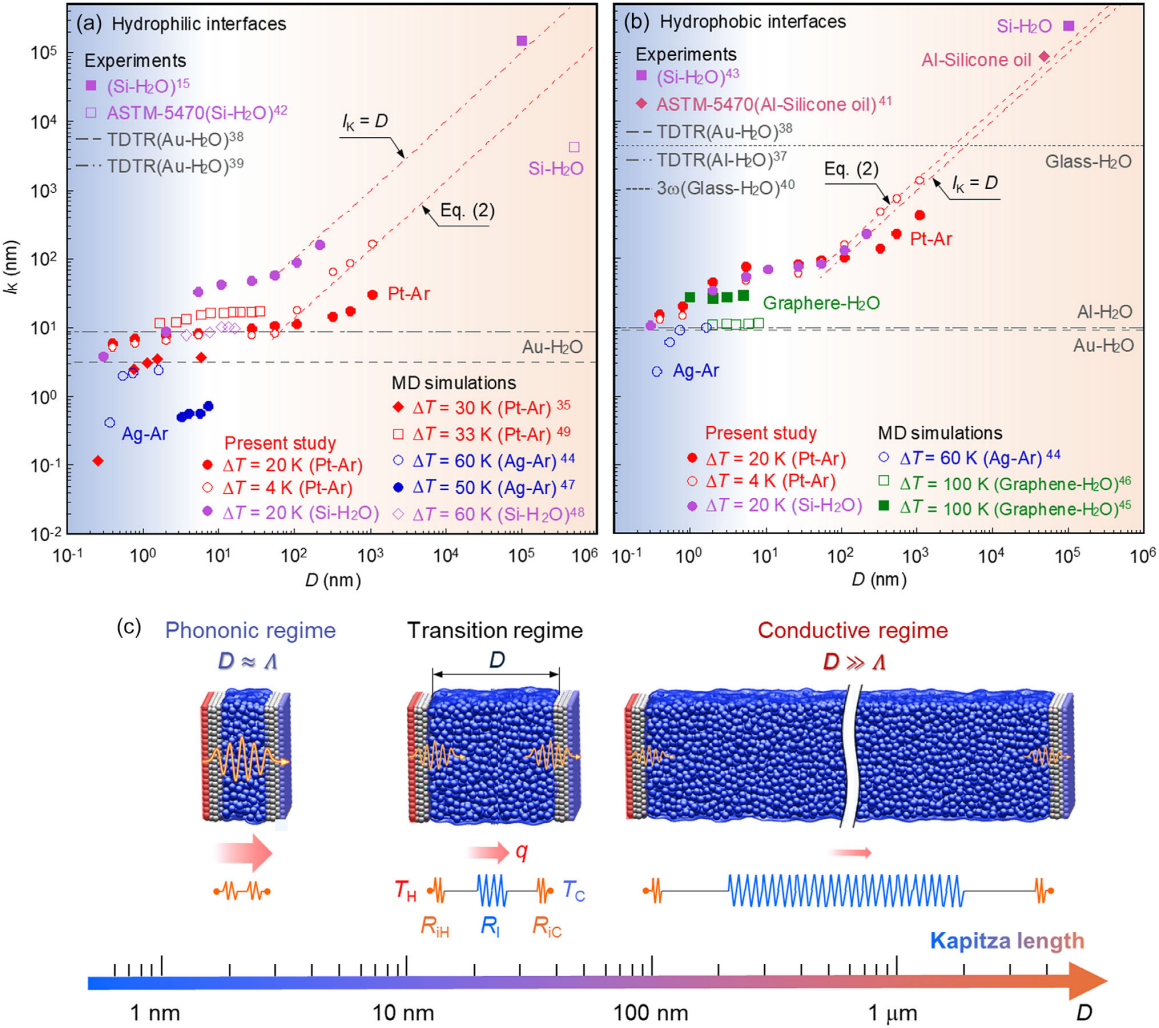
The dependence of Kapitza length *l*_K_ on liquid film thickness *D* under constant Δ*T*: a) hydrophilic cases, b) hydrophobic cases, and c) schematic of phononic, transition, and conductive regimes.

**Figure 10:**
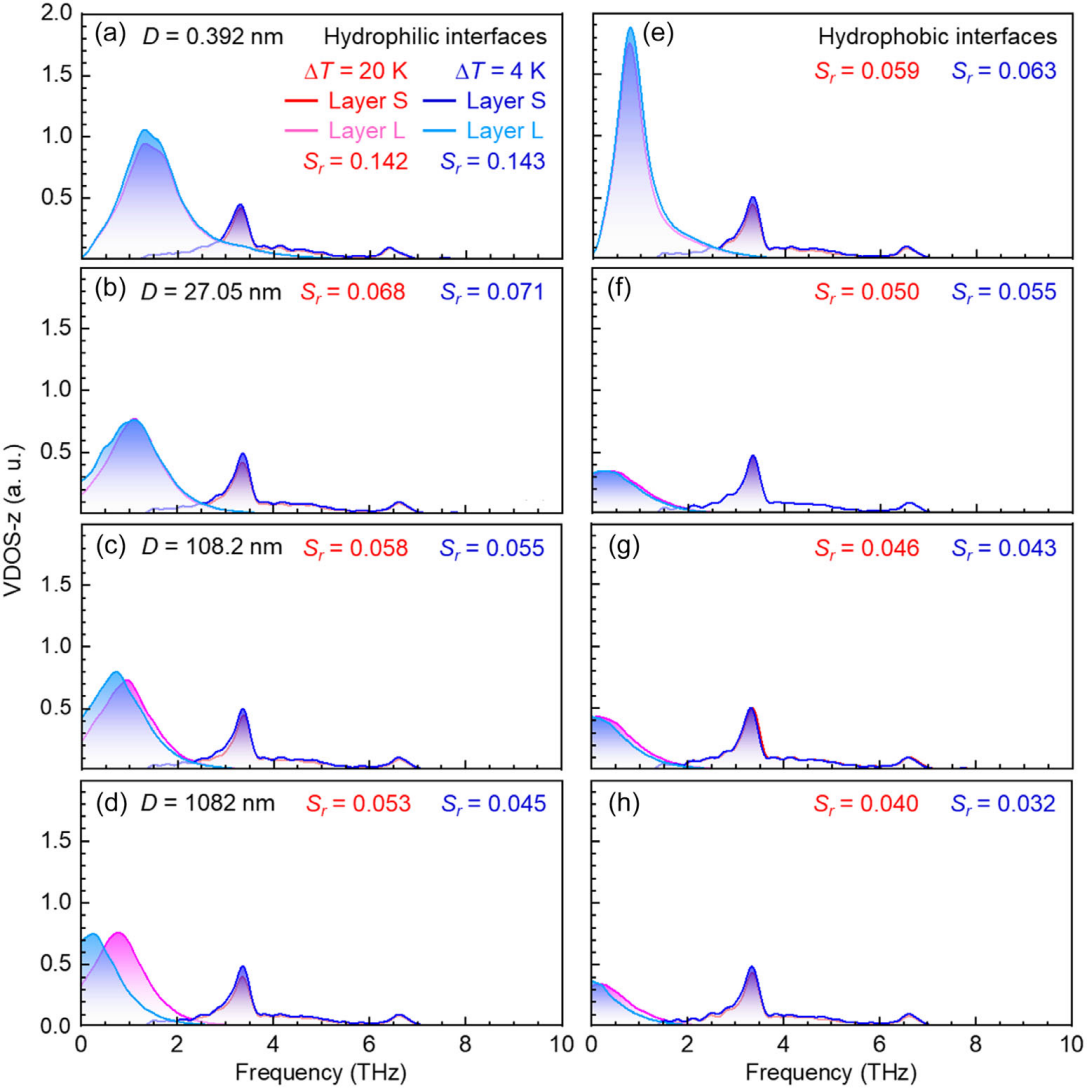
VDOSs of layers S and L under the constant overall temperature difference Δ*T* = 20 and 4 K at *D* = 0.392–1082 nm in a–d) hydrophilic and e–h) hydrophobic cases.

The dependence of *l*_K_ on *D* is illustrated in **Figure** **9**a for hydrophilic cases and Figure 9b for hydrophobic cases. As *D* increases, *l*_K_ becomes larger at both the hydrophilic and hydrophobic Pt–Ar (red dots and circles) and Si–H_2_O (purple dots) interfaces caused by the decreased *q* (or d*T*_l_/d*z*) under constant Δ*T*. This is attributed to a reduction in *Sr* between the interfacial layers S and L with increasing *D*. As shown in **Figure** **10**, the VDOS of the interfacial layer S remains consistent, whereas the peak of the VDOS at the interfacial layer *L* shifts toward the lower frequency band with increasing *D*, resulting in a greater mismatch between layers *S* and *L*. This mismatch is more pronounced in hydrophobic cases, causing larger *l*_K_ at the corresponding interfaces. Moreover, the red dashed lines shown in Figure 9 represent the *l*_K_ values predicted by Equation (2) for *D* greater than 50 nm. In hydrophilic cases, the predicted *l*_K_ is lower than *D* (red dash‐dotted line), which is consistent with the lower thermal resistance at the interface compared to that of the bulk liquid. However, predicted *l*_K_ is higher than *D* in hydrophobic cases, as a result of larger *R*_i_ induced by the significant Δ*T*_i_.

Estimated *l*_K_ based on the data in the literature are presented in Figure 9 for comparison. In Figure 9a, a decrease in *l*_K_ at the Pt–Ar (red diamonds)^[^ ^35^ ^]^ and Ag–Ar (blue circles)^[^ ^44^ ^]^ interfaces with decreasing *D* is shown at *D* = 0.25–5.81 nm, exhibiting a trend consistent with the present data. However, an increase in *D* does not significantly affect *l*_K_ at the Ag–Ar interface (blue dots) for *D* = 3.27–7.35 nm.^[^ ^47^ ^]^ Similarly, *l*_K_ shows minimal dependence on *D* at the Pt–Ar interface (red squares) for *D* = 7.85–35.33 nm,^[^ ^49^ ^]^ aligning with the tendency of present data. At the Si–H_2_O interface, the values of *l*_K_ (purple squares)^[^ ^15^, ^42^ ^]^ estimated from the microscale experiment are of comparable order of magnitude to the predicted results. However, these values are three to four orders of magnitude larger than those obtained via nanoscale simulations (purple diamonds).^[^ ^48^ ^]^ In Figure 9b, the estimated *l*_K_ at the Ag–Ar interface (blue circles)^[^ ^44^ ^]^ decreases as *D* decreases from 1.62 to 0.36 nm, whereas *l*_K_ at the graphene–H_2_O interfaces (green squares)^[^ ^45^, ^46^ ^]^ remains independent of *D* under a Δ*T* of 100 K and *D* = 1–8 nm. The estimated *l*_K_ at the Al–silicone oil (pink diamond)^[^ ^41^ ^]^ and Si–H_2_O (purple squares)^[^ ^43^ ^]^ interfaces using the steady‐state method are of a similar order of magnitude to the predicted results. However, the estimated *l*_K_ obtained using the TDTR method at the Al–H_2_O^[^ ^37^ ^]^ and Au–H_2_O (black dashed and dash‐dotted lines)^[^ ^38^, ^39^ ^]^ interfaces, is considerably smaller than that at the Si–H_2_O interface using the steady‐state method and the glass–H_2_O interface by the 3ω method (black dotted line).^[^ ^40^ ^]^ It is worth noting that the order of magnitudes of *l*_K_ values estimated by NEMD is comparable to that obtained using the TDTR method. This consistency is attributed to the fact that the TDTR method measures the interfacial thermal conductance at the nanoscale, with negligible interfacial thickness.

Consequently, a significant scale effect of *D* on *l*_K_ is observed in Figure 9. The slope of the presented *l*_K_ in the regime of *D* < 10 nm differs from that in the regimes of 10 nm < *D* < 50 nm and *D* > 50 nm at both the hydrophilic and hydrophobic Pt–Ar interfaces. Therefore, three primary regimes are roughly identified for *l*_K_: 1) decrease in *l*_K_ with decreasing *D* for *D* < 10 nm, 2) minimal impact of *D* on *l*_K_ for 10 nm < *D* < 50 nm, and 3) almost linear increase in *l*_K_ with increasing *D* for *D* > 50 nm. In regime (1), the interfacial heat transfer is predominantly governed by phonon transmission across the solid–liquid interface. This can be attributed to the larger area of the phonon spectrum at interfacial liquid layers, as shown in Figure 10a,e. Frank et al. demonstrated that the phonon mean free path *Λ* in a confined liquid with a *D* of 1.02–8.72 nm exceeds that of the bulk liquid owing to the extended relaxation time of solid‐like liquids near the solid–liquid interface.^[^ ^51^ ^]^ The liquid relaxation time approximates the phonon relaxation time *τ* in solid‐like liquids, with a value of *τ* ≈ 0.02 ns. Subsequently, Λ of solid‐like liquid was estimated to be ≈ 13.38 nm, as calculated using $\Lambda =v_{a}\tau$,^[^ ^52^ ^]^ where *v*_*a*_ denotes the average speed of phonons (669.2 m s^−1^). For saturated argon bulk liquid, Λ is ≈ 0.74 nm at a temperature of 110 K, as calculated using $\Lambda =3\lambda_{l}/v_{a}C_{v}\rho_{l}$, where *C*_*v*_ is the heat capacity per unit volume (474.68 J kg^−1 ^K^−1^).^[^ ^53^ ^]^ Considering that Λ falls approximately within the range of 0.74–13.38 nm, which is comparable to *D*, regime (1) is characterized as the phononic regime for the contribution of phonons to interfacial heat transfer. In regime (2), interfacial heat transfer is influenced by both the solid‐like liquid near the solid–liquid interface and the bulk liquid. Given that the phonon mean free path is substantially shorter in a bulk liquid, phonons can only transmit across the solid–liquid interface before dissipating in the bulk liquid. In regime (3), the thermal resistance of the bulk liquid notably increases with *D*, leading to a linear increase in *l*_K_ in accordance with Fourier's law. Thus, interfacial heat transfer in larger‐scale systems is primarily driven by thermal conduction in the bulk liquid. Defining regime (3) as the conductive regime, the regime (2) becomes a transition between the phononic and conductive regimes. Consequently, the regime classification can be summarized as 1) phononic regime (D≈Λ), 2) transition regime, and 3) conductive regime (D≫Λ). Notably, a similar trend in *l*_K_ was found at the Si–H_2_O interface (purple dots) across three regimes. Previous studies have primarily focused on the phononic and transition regimes, as small‐scale systems were conducted to reduce computational costs. As a result, three primary regimes of solid–liquid interfacial heat transfer are dependent on the system scale, rather than interfacial materials. The regime classification is empirical and problem dependent and facilitates an understanding of the mechanisms that influence the scale effect of the Kapitza length on interfacial heat transfer across nano‐ and microscales.

## 3 Conclusions

Kapitza length is of paramount importance for solid–liquid interfacial heat transfer in nano/microelectronics. It is typically estimated under constant heat flux in experiments^[^ ^15^, ^41^, ^42^ ^]^ and constant overall temperature difference in NEMD simulations.^[^ ^47^, ^48^, ^49^ ^]^ In experiments, Kapitza length is generally estimated as a constant for the same surface, irrespective of the heating method. While the steady‐state method employs the constant heat flux, the transient method often lacks information on heat flux and overall temperature difference.^[^ ^37^, ^38^, ^39^, ^40^ ^]^ This leads to the inconsistent Kapitza length among the experimental methods. In the NEMD simulations, nanoscale liquid film commonly subjects to significant overall temperature differences,^[^ ^44^, ^45^, ^46^ ^]^ resulting in considerable heat flux and temperature gradient deviated from the practical conditions. Thus, the knowledge gap between the experiments and simulations exists in understanding Kapitza length at the nano/microscale.

To address this knowledge gap, Kapitza length at the Pt–Ar and Si–H_2_O interfaces was examined under constant heat flux and overall temperature difference in the present NEMD simulations. The findings show that Kapitza length remains nearly constant under constant heat flux, which aligns with the steady‐state method in experiments. However, under constant overall temperature differences, the heat flux across nanoscale liquid film is three orders of magnitude greater than that across microscale liquid film. Additionally, Kapitza length is comparable in magnitude to the liquid film thickness, suggesting that heat flux is a key determinant of Kapitza length. Notably, under constant overall temperature differences, Kapitza length exhibits remarkable scale dependence on the liquid film thickness.

At the solid–liquid interface, the phonon mean free path of solid‐like liquid is ≈10 nm, whereas that in the bulk liquid is much shorter. Therefore, phonon transmission across the interface plays a primary role in the cases of liquid film thicknesses less than 10 nm. Its importance decreases with increasing liquid film thickness owing to phonon dissipation in the bulk liquid. For liquid film thicknesses larger than 50 nm, interfacial heat transfer is dominated by thermal conduction in the bulk liquid. Accordingly, three primary regimes are identified from the scale dependence of Kapitza length on liquid film thickness: phononic, transition, and conductive regimes.

The solid–liquid interfacial resistance, quantified by slip length and Kapitza length, cannot be ignored when these lengths are comparable with hydraulic diameter.^[^ ^54^, ^55^, ^56^, ^57^ ^]^ The coupled effects of slip length and Kapitza length on solid–liquid interfacial heat transfer should be the focus of future work. The related experimental research is desirable to validate the proposed mechanisms and further deepen the understanding of nano/microscale heat transfer phenomena at solid–liquid interfaces.

## 4 Simulation Methods

4.1

4.1.1

##### MD Simulations

The NEMD method^[^ ^58^, ^59^, ^60^, ^61^, ^62^, ^63^ ^]^ was utilized to simulate thermal energy transport at solid–liquid interfaces for varying *D* using an open‐source, large‐scale atomic/molecular massively parallel simulator.^[^ ^64^, ^65^ ^]^ Two distinct simulation systems, Pt–Ar and Si–H_2_O, were employed. The details of the Si–H_2_O system are outlined in Section 6 of the Supporting Information. For the Pt–Ar system, the dimensions of the simulation cell were *L*_*x*_ = 5.55 nm, *L*_*y*_ = 3.85 nm, and *L*_*z*_ ranging from 2.205 to 1084 nm. The distance between the two solid walls varied from 0.392 to 1082 nm. Each solid wall was constructed from face‐centered cubic lattices comprising four layers of 1280 Pt atoms, oriented with the <111> crystal plane facing the liquid film. The liquid films consisted of 190–431 640 Ar atoms, sandwiched between a cooling wall and a heating wall. At *D* = 2–1082 nm, the saturated density of liquid Ar was maintained at ≈1239.6 kg m^−3^ at 110 K. Periodic boundary conditions were applied in the *x*‐ and *y*‐directions.

The Lennard–Jones (LJ) potential $\phi \left(r_{ij}\right)=4\varepsilon \left[{\left(\sigma /r_{ij}\right)}^{12}-{\left(\sigma /r_{ij}\right)}^{6}\right]$ is widely used for solid–solid and liquid–liquid interactions in NEMD simulations,^[^ ^66^, ^67^ ^]^ where *r*_*ij*_ represents the distance between atoms *i* and *j*, *σ* is the length parameter, and *ε* is the energy parameter. The values of these parameters are $\sigma_{l}$ = 3.405 Å and $\varepsilon_{l}$ = 1.67 × 10^−21^ J for the liquid–liquid interaction of Ar, and $\sigma_{s}$ = 2.475 Å and $\varepsilon_{s}$ = 8.35 × 10^−20^ J for the solid–solid interaction of Pt. A modified LJ potential was employed for the solid–liquid interaction

(4) $$\phi_{\text{sl}}\left(r_{ij}\right)=4\alpha \varepsilon_{\text{sl}}\left[{\left(\sigma_{\text{sl}}/r_{ij}\right)}^{12}-\beta {\left(\sigma_{\text{sl}}/r_{ij}\right)}^{6}\right]$$

where $\sigma_{\text{sl}}=\left(\sigma_{s}+\sigma_{l}\right)/2$ and $\varepsilon_{\text{sl}}=\alpha \sqrt{\varepsilon_{s}\varepsilon_{l}}$ were utilized based on the Lorentz–Berthelot combining rule.^[^ ^68^, ^69^ ^]^ In this study, the strength of the molecular interactions was calibrated using factors *α* and *β*, derived from contact angle measurements reported in previous studies.^[^ ^70^ ^]^ Specifically, *α* was set to a constant value $\alpha =\sqrt{\varepsilon_{l}/\varepsilon_{s}}$ of 0.14. The hydrophilic and hydrophobic interactions were characterized using *β* values of 1.0 and 0.5, respectively.^[^ ^19^ ^]^

All the simulations employed a cutoff radius of 3.5 $\sigma_{l}$, which was selected to ensure accuracy using a spherically truncated and shifted potential. The equations of motion were integrated using the velocity Verlet algorithm with a time step of 5 fs. Initially, the simulated systems were operated in a canonical ensemble (NVT) at 100 K for 10 ns. Subsequently, for a constant Δ*T* using the velocity scaling method, the thermostat was removed from the liquid, and the temperature of the heating thermostat was increased to 120 K, whereas the cooling thermostat was maintained at 100 K. This setup resulted in a temperature difference Δ*T* of 20 K between the thermostats. To achieve a smaller Δ*T* of 4 K, the temperature of the cooling thermostat subsequently increased to 116 K. For constant *q* condition, the heating and cooling thermostats added and subtracted energy at *q* values of 2, 8, or 20 MW m^−2^. The NEMD simulations were conducted over a 500 ns interval to attain a steady state in the NVE ensemble.

##### Data Collection

After ensuring steady‐state conditions for 25 ns, data were collected to determine the density, temperature, and heat flux. The heat flux is expressed as q=∂E/∂t/A, where *A* represents the cross‐sectional area of the *x‐y* plane and ∂E/∂t is the cumulative energy exchange in the heating (*E*_H_) or cooling (*E*_C_) thermostat. The thermal conductivity of the bulk liquid was calculated using Fourier's law, $\lambda_{l}=q/(dT_{l}/dz)$, where d*T*_l_/dz is the temperature gradient determined by linear fitting across the liquid region. Figure S9, Supporting Information, indicates that the thermal conductivity *λ*_l_ remains consistent for *D* > 2 nm, aligning with a value of 0.0972 W m^−1^ K^−1^.^[^ ^71^ ^]^ However, for *D* = 0.392–0.784 nm where the liquid film is only 1–2 molecular layers thick, *λ*_l_ calculated using the Green–Kubo method yielded a higher value compared with that of the bulk liquid. The interfacial thermal resistance *R*_i_ was determined as the average of the thermal resistances at the heating *R*_iH_ and cooling *R*_iC_ interfaces.

##### Vibration Density of States

The VDOSs of the interfacial solid and liquid layers were calculated using the velocities of all interfacial atoms by obtaining the Fourier transform of the velocity autocorrelation function for a period of 20 000 time steps^[^ ^63^, ^72^ ^]^

(5) $$\text{VDOS}(\omega )=\int_{0}^{\infty }\left\langle \vec{v}\left(t\right)\vec{v}\left(0\right)\right\rangle e^{-i\omega t}dt$$

where *ω* is the angular frequency and *v*(*t*) is the atomic velocity at time *t*. The overlap of the VDOSs *Sr* can be utilized to assess the degree of match between the VDOS profiles, serving as a critical metric for elucidating phonon–phonon coupling.^[^ ^73^, ^74^ ^]^ To quantify the degree of phonon matching between the interfacial solid and the liquid layers, *Sr* was calculated as the ratio of overlap to the total area of the VDOS integrated over the angular frequency range from zero to infinity

(6) $$S_{r}=\frac{\int_{0}^{\infty }\min\left\{V_{1}\left(\omega \right),V_{2}\left(\omega \right)\right\}d\omega }{\int_{0}^{\infty }\max\left\{V_{1}\left(\omega \right),V_{2}\left(\omega \right)\right\}d\omega }$$

where *V*_1_ and *V*_2_ represent the VDOSs of interfacial solid and liquid layers, respectively.

##### Statistical Analysis

All data were analyzed and processed using KaleidaGraph 4.5. In each simulation case, the data was collected in a steady state over a 25 ns period. Representative temperature results were evaluated at 5 ns intervals to calculate the average temperature throughout the 25 ns period. The SE and RSE of local temperature were less than 0.062 K and 0.052% in the Pt–Ar systems, as well as less than 0.01 and 0.003% in the Si–H_2_O systems, thus confirming the reliability of the simulation results.

## Conflict of Interest

The authors declare no conflict of interest.

## Author Contributions

**Wentao Chen**: data curation (lead); formal analysis (lead); investigation (lead); methodology (equal); validation (equal); writing—original draft (lead). **Gyoko Nagayama**: conceptualization (lead); funding acquisition (lead); methodology (equal); validation (equal); writing—review & editing (lead).

## Supporting information

Supplementary Material

## Data Availability

The data that support the findings of this study are available from the corresponding author upon reasonable request.
