# Supplementary material for: Kapitza Length at Solid–Liquid Interface: From Nanoscale to Microscale
Source: Small Sci. 2025 Mar 8;5(6):2400626. doi: 10.1002/smsc.202400626 (PMC12168602; doi:10.1002/smsc.202400626)
Supplement: Supplementary file 1 — Supplementary Material [file SMSC-5-2400626-s001.pdf]

Supporting Information for

Kapitza Length at Solid–Liquid Interface: From Nanoscale to Microscale

*Wentao Chen, Gyoko Nagayama**

Department of Mechanical Engineering, Kyushu Institute of Technology,
Kitakyushu, Fukuoka 804-8550, Japan
E-mail: nagayama.gyoko725@mail.kyutech.jp

1. Density and temperature profiles

The solid–liquid interaction can result in the formation of solid–like liquid layers on the solid surface, which is critical in determining the interfacial thermal resistance [1,2]. Consequently, density profiles for channel width ranging from 5.41 to 324.60 nm were calculated under ΔT = 20 K and 4 K in the hydrophilic and hydrophobic Pt–Ar systems, as shown in Figs. S1 and S2. In hydrophilic cases, the thickness of density profiles in the z-direction at the heating and cooling regions remain consistent, as shown in Fig. S1. The liquid atoms are orderly distributed in the interfacial region, where the local density deviates from the bulk value. With an increase in D, the peak density value of the liquid layer adjacent to cooling solid surface is higher than

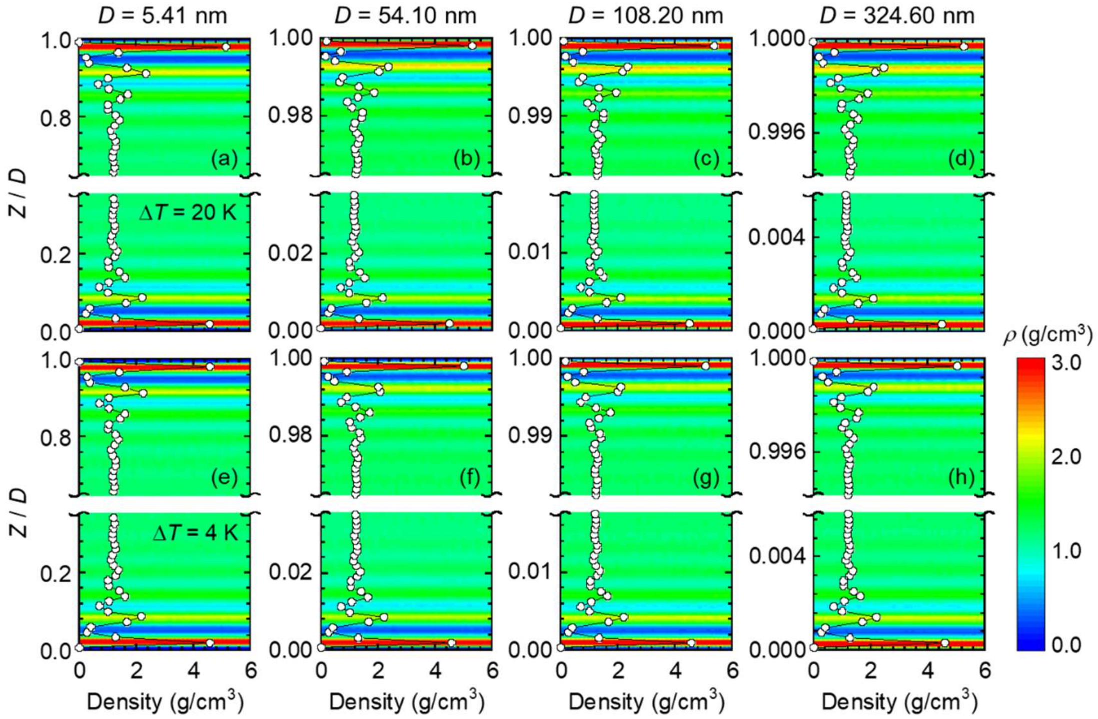

Fig. S1 Density profiles as liquid film thickness D = 5.41–324.60 nm under the overall temperature difference **a–d** ΔT = 20 K and **e–h** ΔT = 4 K in hydrophilic Pt–Ar systems.

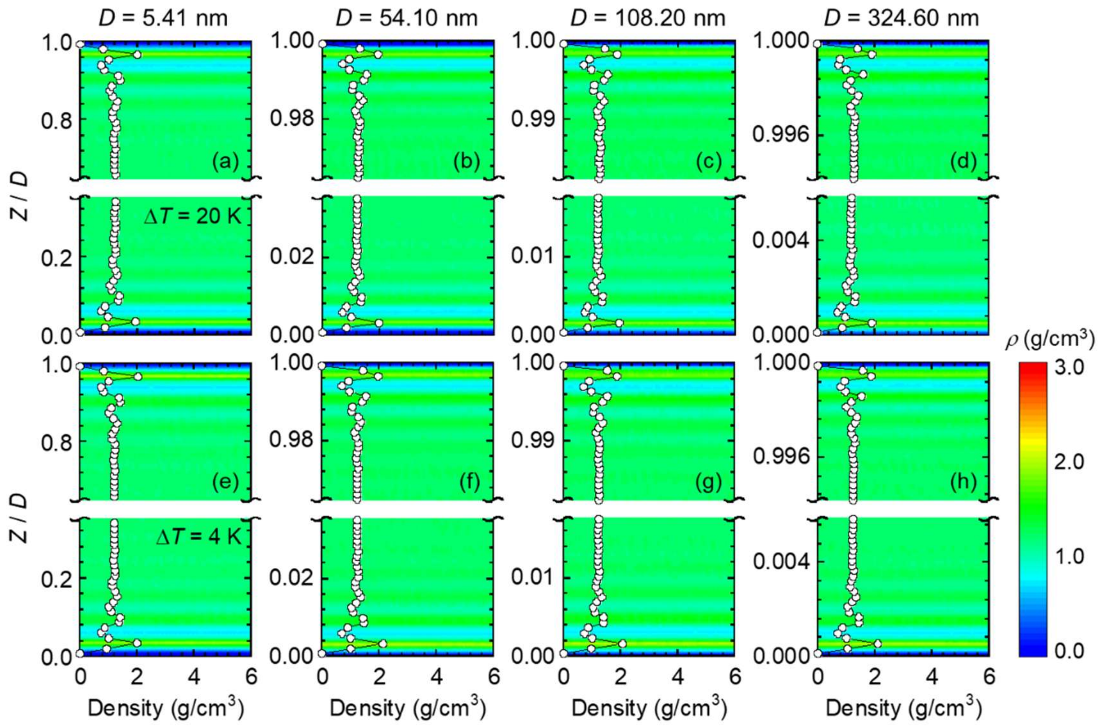

Fig. S2 Density profiles as liquid film thickness D = 5.41–324.60 nm under the overall temperature difference **a–d** ΔT = 20 K and **e–h** ΔT = 4 K in hydrophobic Pt–Ar systems.

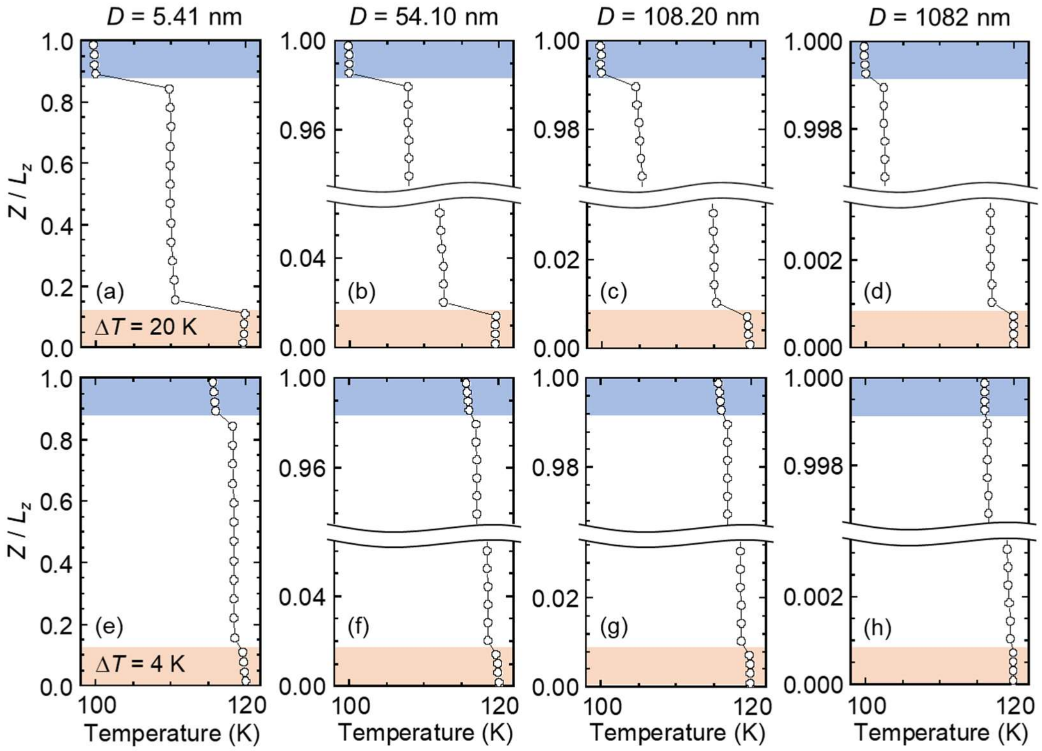

Fig. S3 Temperature profiles as D = 5.41–1082 nm under the overall temperature difference **a–d** ΔT = 20 K and **e–h** ΔT = 4 K in hydrophobic Pt–Ar systems.

that to heating solid surface, due to stronger interactions with the cooling solid surface. Specifically, the thickness of cooling interfacial region δ_i is approximately 1.80 nm in the

hydrophilic cases, featuring several three orderly liquid layers at various D under $\Delta T = 20$ K. The liquid density distribution is nearly consistent at various D in the heating or cooling interfacial region. For hydrophobic cases, Fig. S2 shows similar results for density distribution within the heating or cooling interfacial regions as D increases. However, δ_l is nearly 1.38 nm, which is smaller than that in hydrophilic cases. This discrepancy indicates that the liquid density in the interfacial region is dependent on solid–liquid interactions, rather than D. This observation is in agreement with a previous study on heat transfer across graphene–H_2O interface [3].

2. Error analysis of temperature

All simulation data were collected in a steady state over a 25 ns period. Temperatures were evaluated every 5 ns to calculate the average temperature throughout the 25 ns period. Tables S1 and S2 present error analysis examples for the local temperatures at the interfacial liquid layers adjacent to the heating and cooling walls ($\Delta T = 4$ K; $D = 5.41$, 108.2, and 1082 nm, in hydrophilic cases). The temperature fluctuations over time were minimal, and both the standard error (SE) and relative standard error (RSE) of the temperature were insignificant.

Table S1 Error analysis for temperatures at the interfacial liquid layer adjacent to the heating solid wall ($\Delta T = 4$ K, hydrophilic Pt–Ar cases).

Time period (ns)	$D = 5.41$ nm	$D = 108.2$ nm	$D = 1082$ nm
0–5	118.509	119.477	119.594
5–10	118.538	119.500	119.525
10–15	118.456	119.588	119.551
15–20	118.600	119.607	119.558
20–25	118.602	119.566	119.601
$\bar{T}$ (K)	118.541	119.548	119.566
SE (K)	0.062	0.056	0.032
RSE (%)	0.052	0.047	0.026

Table S2 Error analysis for temperatures at the interfacial liquid layer adjacent to the cooling solid wall (ΔT = 4 K, hydrophilic Pt–Ar cases).

Time period (ns)	D = 5.41 nm	D = 108.2 nm	D = 1082 nm
0–5	117.409	116.471	116.442
5–10	117.402	116.440	116.412
10–15	117.436	116.441	116.479
15–20	117.443	116.433	116.442
20–25	117.410	116.454	116.386
$\bar{T}$ (K)	117.420	116.448	116.432
SE (K)	0.018	0.015	0.035
RSE (%)	0.016	0.013	0.030

3. Temperature difference at solid–liquid interface and temperature gradient of liquid

Figure S4 presents the detailed temperature profiles in a larger hydrophilic Pt–Ar simulation system with ΔT of 4 K and D of 1.082 μm. The local temperature of the liquid was measured using 2424 bins along the z-direction, each with a width of 0.446 nm. The temperatures of both the local liquid and solid phases correspond well with the linear fit, exhibiting a goodness of fit R^2 of 0.989 for the bulk liquid, 0.995 for the heating wall, and 0.993 for the cooling wall. Tables S3 and S4 provide error analysis for temperatures at the solid–liquid interfaces adjacent to the heating and cooling walls, as well as temperature gradients in the bulk liquid and solid walls. The extrapolated interfacial temperatures, T_{wi} and T_{li} from the linear fits, and the difference ($\Delta T_i = T_{wi} - T_{li}$) show minimal variation over time. Both the SE and RSE for ΔT_i are below 0.01 and 1.7%, respectively.

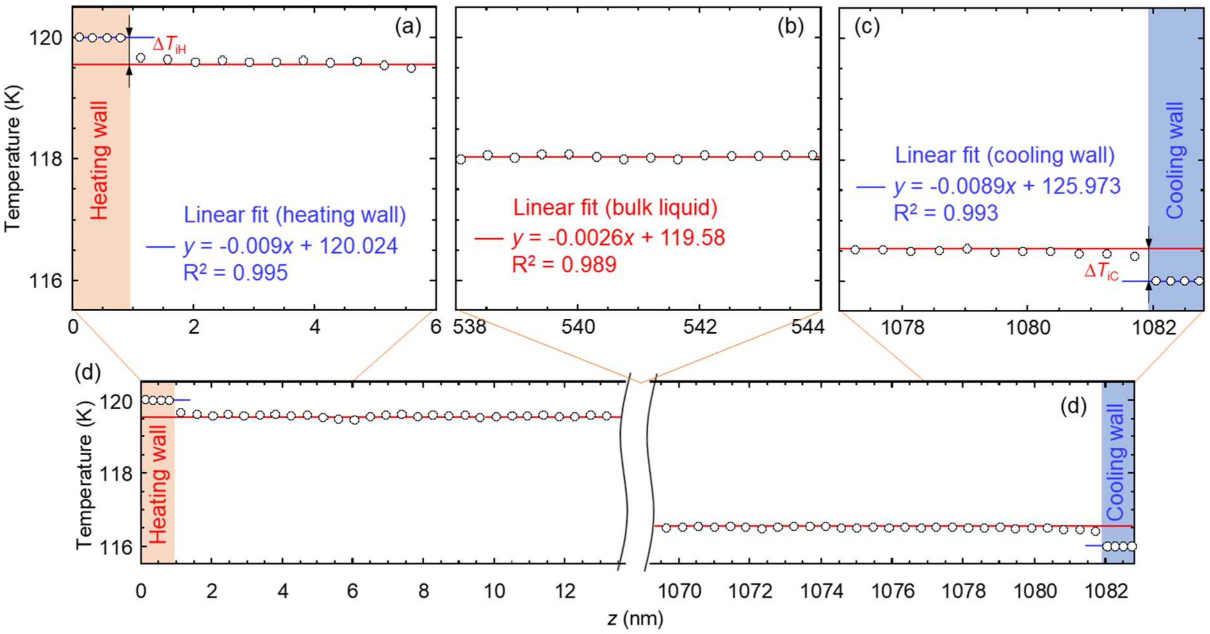

Fig. S4 Temperature profiles for a hydrophilic Pt–Ar simulation system with $D = 1.082$ μm at $\Delta T = 4$ K: **a** solid–liquid interface near the heating wall, **b** bulk liquid, **c** solid–liquid interface near the cooling wall, and **d** overall system.

Table S3 Error analysis of temperatures at the hydrophilic Pt–Ar interface near the heating wall for a simulation system with $D = 1.082$ μm at $\Delta T = 4$ K. The analysis includes temperature gradients in the solid wall $\mathrm{d}T_s/\mathrm{d}z$ and bulk liquid $\mathrm{d}T_l/\mathrm{d}z$, and the interfacial temperatures (T_{wi} and T_{li}) from the linear temperature extrapolation.

Time period (ns)	$\mathrm{d}T_s/\mathrm{d}z$ (K/nm)	$\mathrm{d}T_l/\mathrm{d}z$ (K/nm)	T_{wi} (K)	T_{li} (K)	ΔT_i (K)	G (MW m^{-2} K^{-1})
0–5	–0.009000	–0.002604	120.012	119.584	0.428	0.596
5–10	–0.009004	–0.002602	120.017	119.586	0.431	0.592
10–15	–0.009002	–0.002609	120.028	119.582	0.446	0.572
15–20	–0.009000	–0.002596	120.008	119.582	0.426	0.599
20–25	–0.009008	–0.002601	120.014	119.578	0.436	0.585
average	–0.009003	–0.002602	120.016	119.582	0.434	0.589
SE	2.993×10^{-6}	4.224×10^{-6}	0.007	0.003	0.007	0.010
RSE (%)	0.033	0.162	0.006	0.002	1.648	1.627

7

Table S4 Error analysis of temperatures at the hydrophilic Pt–Ar interface near the cooling wall for a simulation system with D = 1.082 μm at ΔT = 4 K. This analysis includes temperature gradients in the solid wall $\mathrm{d}T_s/\mathrm{d}z$ and bulk liquid $\mathrm{d}T_\mathrm{l}/\mathrm{d}z$, as well as interfacial temperatures (T_wi and T_li) derived from the linear temperature extrapolation.

Time period (ns)	$\mathrm{d}T_\mathrm{s}/\mathrm{d}z$ (K/nm)	$\mathrm{d}T_\mathrm{l}/\mathrm{d}z$ (K/nm)	T_wi (K)	T_li (K)	ΔT_i (K)	G (MW m^{-2} K^{-1})
0–5	–0.008900	–0.002604	116.342	116.766	0.424	0.602
5–10	–0.008906	–0.002602	116.333	116.770	0.437	0.583
10–15	–0.008903	–0.002609	116.323	116.759	0.436	0.586
15–20	–0.008901	–0.002596	116.331	116.773	0.442	0.578
20–25	–0.008899	–0.002601	116.336	116.763	0.427	0.597
average	–0.008902	–0.002602	116.333	116.766	0.433	0.589
SE	2.482×10^{-6}	4.224×10^{-6}	0.006	0.006	0.007	0.009
RSE (%)	0.028	0.162	0.005	0.004	1.534	1.540

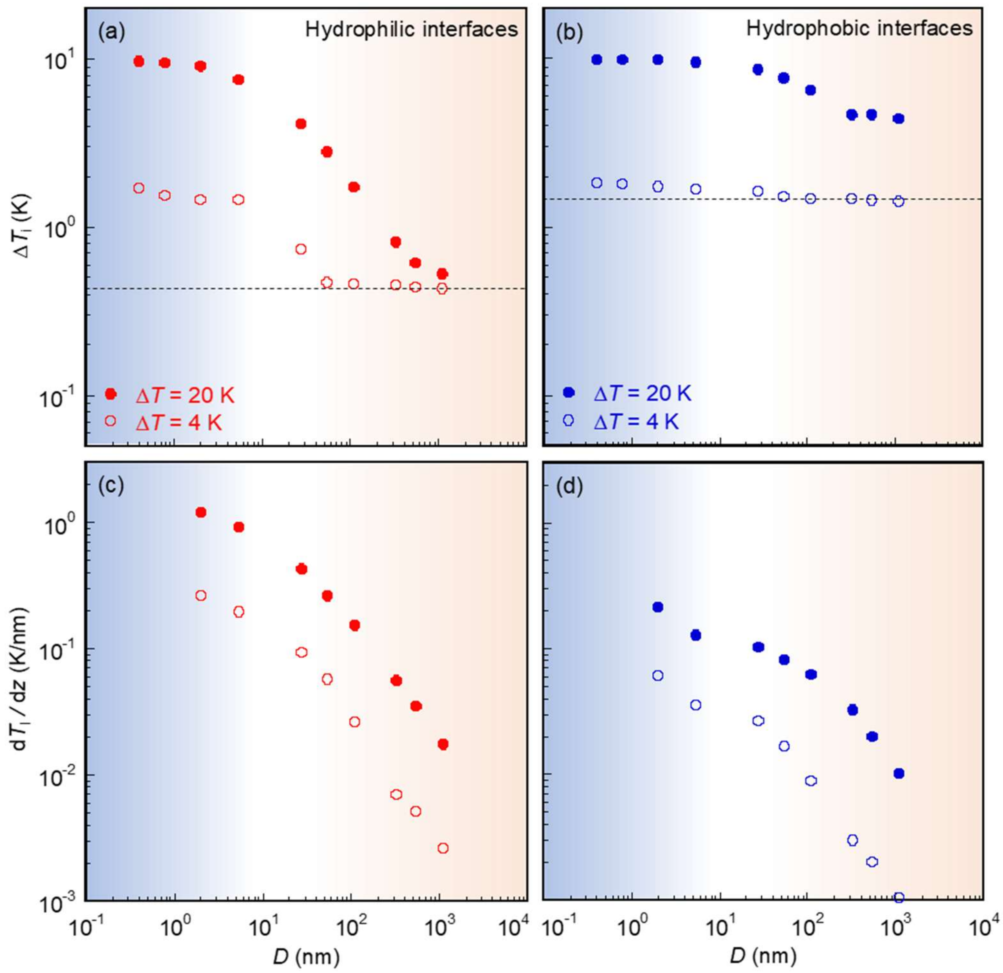

Fig. S5 a–b Temperature difference at Pt–Ar interface ΔT_i and **c–d** temperature gradient of liquid dT_l/dz versus liquid film thickness D of 0.392 nm–1.082 μm under the overall temperature difference $\Delta T = 20$ K and 4 K in hydrophilic and hydrophobic cases.

4. Interfacial temperature effects on thermal conductance

Tables S5 and S6 document the interfacial temperatures at the heating/cooling walls and the thermal conductance G for various D. The solid interfacial temperatures T_{wi} at both heating and cooling walls remain nearly constant, whereas the liquid interfacial temperatures T_{li} show a dependence on D. At the heating wall, T_{li} increases, but it decreases at the cooling wall with increasing D when $D < 54.1$ nm, and stabilizes for $D > 54.1$ nm, as illustrated in Fig. S6. Furthermore, Figs. S7 and S8 depict the relationships between T_{wi} or ΔT_i and G. G is largely independent of T_{wi} but exhibits a positive correlation with ΔT_i, which corresponds to the phononic, transition, and conductive regimes.

Table S5 Interfacial temperatures at heating/cooling walls and thermal conductance for D = 0.392–1082 nm at ΔT = 4 K in hydrophilic Pt–Ar cases.

D (nm)	Heating wall			Cooling wall			G (MW m^{-2} K^{-1})
	T_{wi} (K)	T_{li} (K)	ΔT_i (K)	T_{wi} (K)	T_{li} (K)	ΔT_i (K)	
0.392	119.846	117.935	1.911	116.040	117.935	1.895	30.320
0.784	119.849	118.077	1.772	116.096	117.831	1.735	16.811
2.000	119.866	118.178	1.688	116.045	117.718	1.673	15.255
5.410	119.958	118.293	1.665	115.966	117.420	1.454	12.444
27.05	119.998	119.236	0.762	115.988	116.714	0.726	12.266
54.10	120.003	119.530	0.473	115.986	116.452	0.466	11.789
108.2	120.014	119.548	0.466	115.987	116.448	0.461	5.481
324.6	120.023	119.562	0.461	115.988	116.435	0.447	1.480
541.0	120.013	119.564	0.449	115.984	116.421	0.437	1.126
1082	120.016	119.582	0.434	115.974	116.406	0.432	0.589

Table S6 Interfacial temperatures at heating/cooling walls and thermal conductance for D = 0.392–1082 nm at ΔT = 4 K in hydrophobic Pt–Ar cases.

D (nm)	Heating wall			Cooling wall			G (MW m^{-2} K^{-1})
	T_{wi} (K)	T_{li} (K)	ΔT_i (K)	T_{wi} (K)	T_{li} (K)	ΔT_i (K)	
0.392	119.842	118.021	1.821	116.203	118.021	1.818	8.906
0.784	119.855	118.042	1.813	116.186	117.962	1.776	6.834
2.000	119.854	118.101	1.753	116.184	117.905	1.721	3.451
5.410	119.853	118.124	1.729	116.172	117.832	1.660	2.046
27.05	119.851	118.224	1.627	116.078	117.704	1.626	1.581
54.10	119.855	118.322	1.533	116.027	117.515	1.488	1.074
108.2	119.874	118.402	1.472	115.998	117.465	1.467	0.602
324.6	119.883	118.423	1.460	115.994	117.458	1.464	0.200
541.0	119.939	118.496	1.443	115.988	117.421	1.433	0.131
1082	119.946	118.512	1.434	115.973	117.403	1.430	0.070

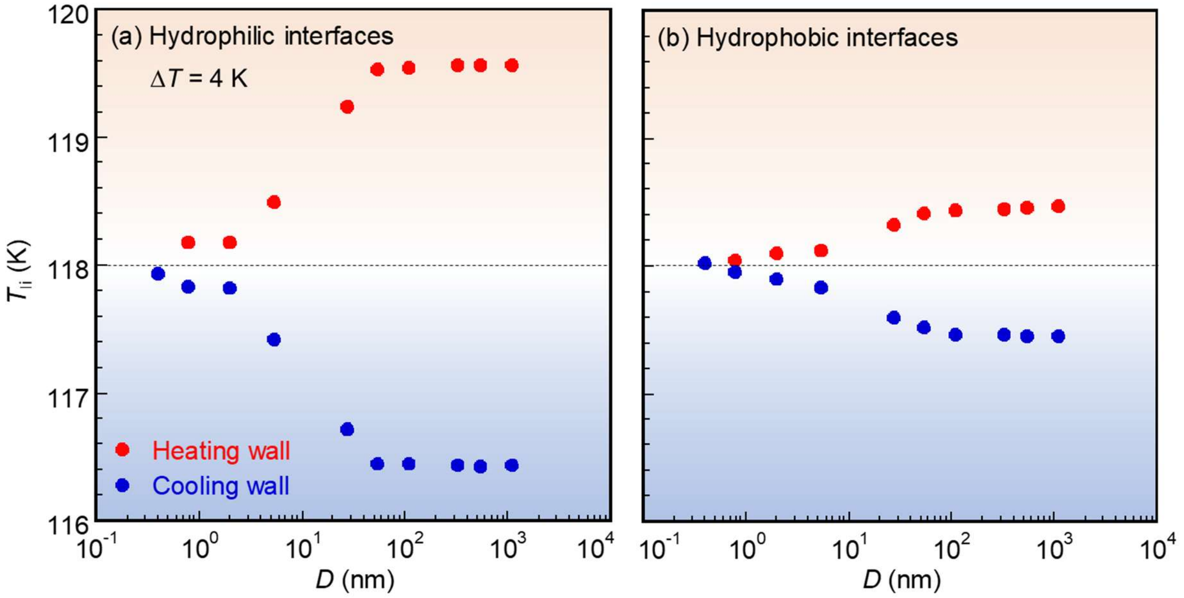

Fig. S6 Temperature dependence at the interfacial liquid layer adjacent to heating and cooling walls T_{li} on D at ΔT = 4 K: **a** hydrophilic Pt–Ar interfaces, **b** hydrophobic Pt–Ar interfaces.

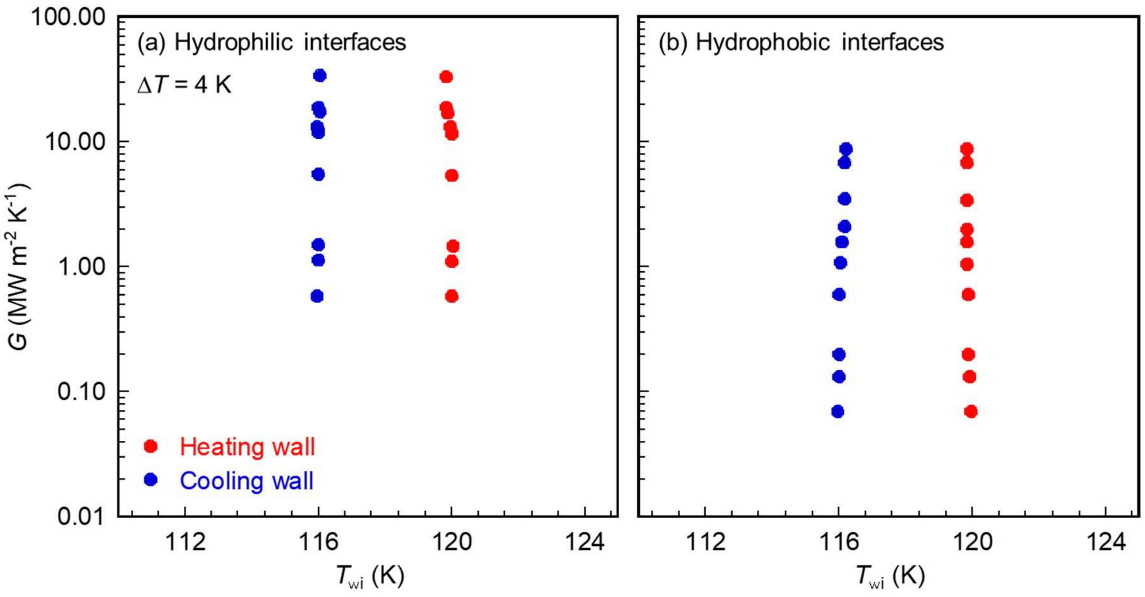

Fig. S7 Interfacial thermal conductance G versus the interfacial solid temperature T_{wi} at ΔT = 4 K: **a** hydrophilic Pt–Ar interfaces, and **b** hydrophobic Pt–Ar interfaces.

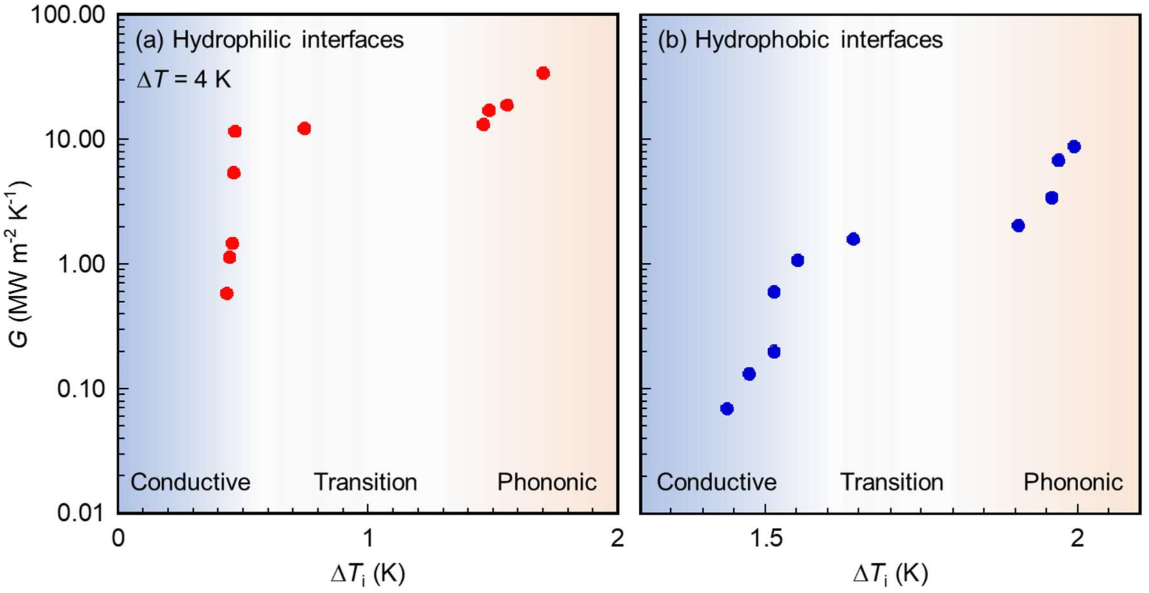

Fig. S8 Dependence of interfacial thermal conductance G on interfacial temperature difference ΔT_i across conductive, transition, and phononic regimes at $\Delta T = 4$ K: **a** hydrophilic Pt–Ar interfaces, and **b** hydrophobic Pt–Ar interfaces.

5. Thermal conductivity of liquid film

Fourier's law was applied to calculate the λ_l for the liquid film in a range of $D = 2$–1082 nm, which met the saturated liquid density. For mono and double liquid layers sandwiched between two solid walls at $D = 0.392$–0.784 nm, the thermal conductivity of the liquid film λ_l was calculated using the Green–Kubo method [4,5]:

$$\lambda_l = \frac{V}{3k_B T^2} \int_0^{\infty} \langle J(0) \cdot J(t) \rangle dt, \tag{S1}$$

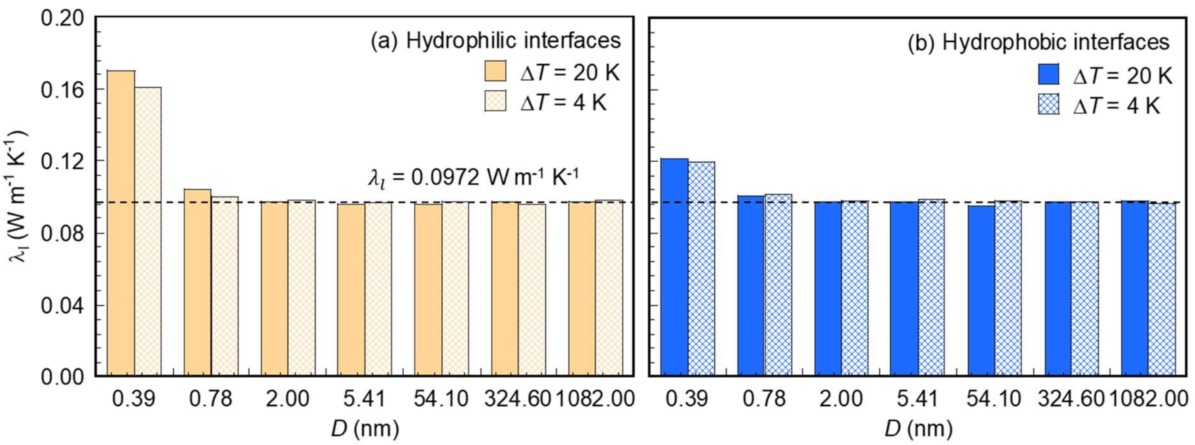

Fig. S9 Thermal conductivity of liquid film as $D = 0.39$ nm to 1.082 μm under $\Delta T = 20$ K and 4 K in: **a** hydrophilic Pt–Ar cases, and **b** hydrophobic Pt–Ar cases. The dotted line represents the property of themal conductivity of liquid Ar at 110 K.

where k_B is the Boltzmann constant, T is the temperature, V is the volume of the calculated region, and J is the heat flux. Figure S9 demonstrates that λ_l approaches a constant value for D = 2–1082 nm and is higher than bulk liquid for D = 0.392–0.784 nm.

6. Thermal conductivity of interfacial liquid layers

The thermal conductivity λ_i of interfacial liquid layers with thickness of 1.38 nm adjacent to the cooling wall at $\Delta T = 20$ K and $D = 5.41$ nm was calculated utilizing the Green–Kubo method. Figure S10 shows that λ_i of the hydrophilic case is greater than that of the hydrophobic case, attributed to more ordered interfacial liquid layers with higher density.

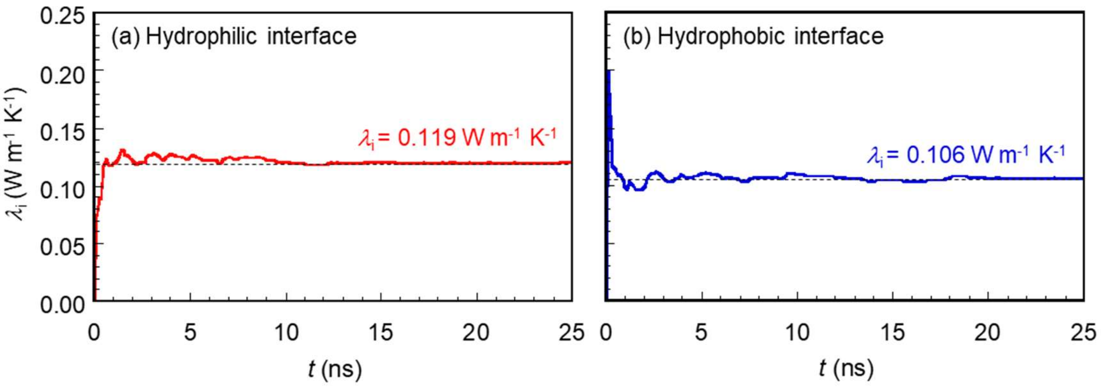

Fig. S10 Thermal conductivity λ_i of interfacial liquid layers with thickness of 1.38 nm adjacent to the cooling wall at ΔT = 20 K and D = 5.41 nm in (a) hydrophilic and (b) hydrophobic Pt–Ar cases.

7. Effects of interfacial and bulk thermal resistance on heat transfer mechanisms

The surface wettability can significantly influence the contributions of interfacial and bulk thermal resistance to heat transfer mechanisms. In hydrophilic cases, Fig. S11 demonstrates an inverse relationship between D and R_{iHC}/R_{total}, while the ratio R_b/R_{total} becomes larger with increasing D. At $D < 10$ nm (phononic regime), the ratio R_{iHC}/R_{total} substantially exceeds R_b/R_{total}, indicating the predominance of interfacial thermal resistance in heat transfer. At $D > 10$ nm (transition and conductive regimes), the effect of R_b on interfacial heat transfer becomes increasingly pronounced. In hydrophobic cases, at $D \leq 108.2$ nm, the ratio R_{iHC}/R_{total} is substantially larger than R_b/R_{total}, attributed to the weak solid–liquid interactions. Conversely, R_b plays a more significant role in the interfacial heat transfer at $D > 108.2$ nm.

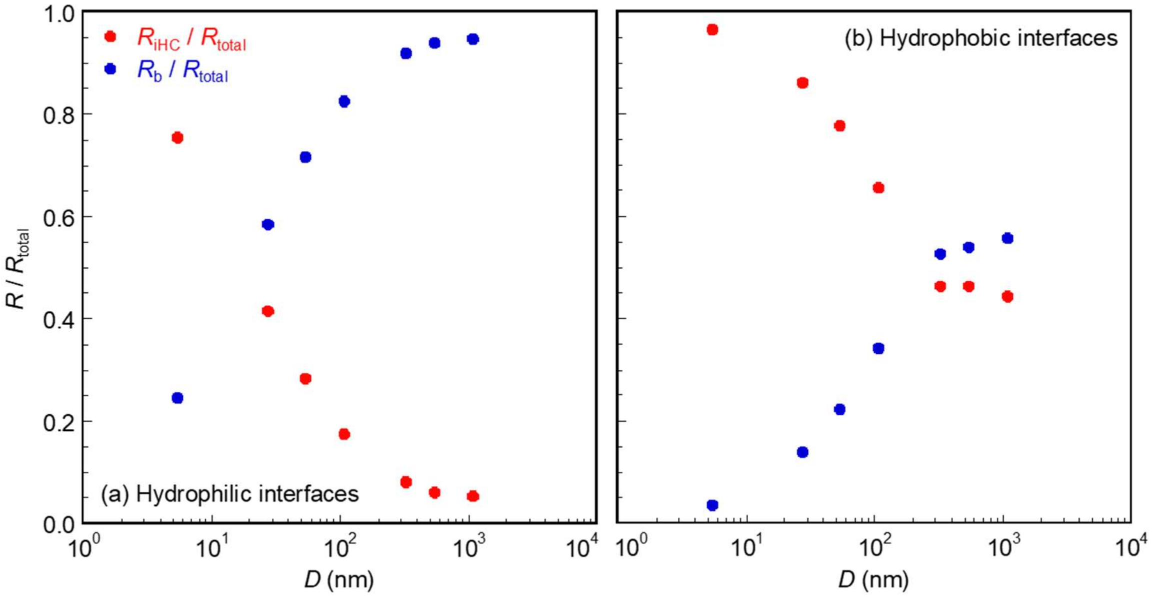

Fig. S11 The ratios R_{iHC}/R_{total} and R_b/R_{total} versus liquid film thickness D under an overall temperature difference of $\Delta T = 20$ K in (a) hydrophilic and (b) hydrophobic cases.

Table S7 Simulation results for hydrophilic Pt–Ar cases.

D (nm)	ΔT_i (K)	$-dT_l/dz$ (K/nm)	λ_l (W m^{-1} K^{-1})	q (MW m^{-2})	G (MW m^{-2} K^{-1})	l_K (nm)
ΔT = 20 K						
0.392	9.685	–	0.1704	278.044	28.709	5.935
0.784	9.558	–	0.1040	136.177	14.247	6.947
2.00	9.185	1.1880	0.0974	115.752	12.602	7.732
5.41	7.540	0.9096	0.0962	87.490	11.604	8.289
27.05	4.152	0.4324	0.0969	41.892	10.090	9.602
54.10	2.829	0.2651	0.0961	25.944	9.170	10.672
108.20	1.750	0.1525	0.0977	14.900	8.515	11.474
324.60	0.814	0.0566	0.0973	5.525	6.789	14.378
541.00	0.614	0.0347	0.0975	3.383	5.513	17.684
1082.0	0.532	0.0175	0.0974	1.704	3.201	30.429
ΔT = 4 K						
0.392	1.903	–	0.1613	57.698	30.320	5.320
0.784	1.754	–	0.1002	29.479	16.811	5.960
2.00	1.681	0.2625	0.0977	25.637	15.255	6.933
5.41	1.560	0.1983	0.0968	19.407	12.444	7.381
27.05	0.744	0.0932	0.0979	9.126	12.266	7.934
54.10	0.469	0.0569	0.0976	5.535	11.789	8.099
108.20	0.463	0.0261	0.0973	2.540	5.481	17.247
324.60	0.454	0.0070	0.0960	0.672	1.480	62.786
541.00	0.443	0.0051	0.0978	0.499	1.126	84.047
1082.0	0.433	0.0026	0.0982	0.255	0.589	166.624

Table S8 Simulation results for hydrophobic Pt–Ar cases.

D (nm)	ΔT_i (K)	$-dT_l/dz$ (K/nm)	λ_l (W m^{-1} K^{-1})	q (MW m^{-2})	G (MW m^{-2} K^{-1})	l_K (nm)
ΔT = 20 K						
0.392	9.997	—	0.1214	78.865	7.889	15.389
0.784	9.928	—	0.1005	61.627	4.888	20.562
2.00	9.851	0.2170	0.0971	21.064	2.138	45.397
5.41	9.655	0.1277	0.0976	12.470	1.292	75.604
27.05	8.614	0.1025	0.0978	10.023	1.164	84.036
54.10	7.776	0.0822	0.0964	7.928	1.019	94.605
108.20	6.570	0.0634	0.0971	6.155	0.937	103.629
324.60	4.644	0.0330	0.0973	3.210	0.691	140.730
541.00	4.631	0.0199	0.0965	1.915	0.414	233.278
1082.0	4.428	0.0103	0.0977	1.007	0.227	429.874
ΔT = 4 K						
0.392	1.820	—	0.1197	16.204	8.906	13.441
0.784	1.795	—	0.1014	12.264	6.834	14.838
2.00	1.737	0.0612	0.0979	5.994	3.451	31.994
5.41	1.695	0.0355	0.0977	3.467	2.046	53.633
27.05	1.627	0.0266	0.0966	2.571	1.581	61.663
54.10	1.511	0.0166	0.0979	1.622	1.074	93.577
108.20	1.470	0.0090	0.0983	0.885	0.602	168.122
324.60	1.462	0.0030	0.0976	0.293	0.200	504.367
541.00	1.438	0.0020	0.0967	0.188	0.131	755.141
1082.0	1.432	0.0010	0.0968	0.101	0.070	1382.077

8. Simulation method of Si–H_2O system

The dimensions of the Si–H_2O simulation cell were L_x = 2.82 nm, L_y = 2.82 nm, and L_z = 8.10–224.2 nm, with the distance between the two silicon walls D ranging from 0.3 to 216.4 nm, as illustrated in Fig. S12. Each silicon wall consisted of 1400 silicon atoms, arranged in a diamond cubic structure with the (0,0,1) crystal plane oriented toward the water. The water films, comprising 70–52560 molecules, were positioned between a cooling wall (maintained at 290 K) and a heating wall (maintained at 310 K). These configurations were based on the saturated liquid density of approximately 0.997 kg/m^3 at 300 K. The temperatures of the heating and cooling layers were controlled using the Langevin thermostat. Periodic boundary conditions were applied in the *x*- and *y*- directions.

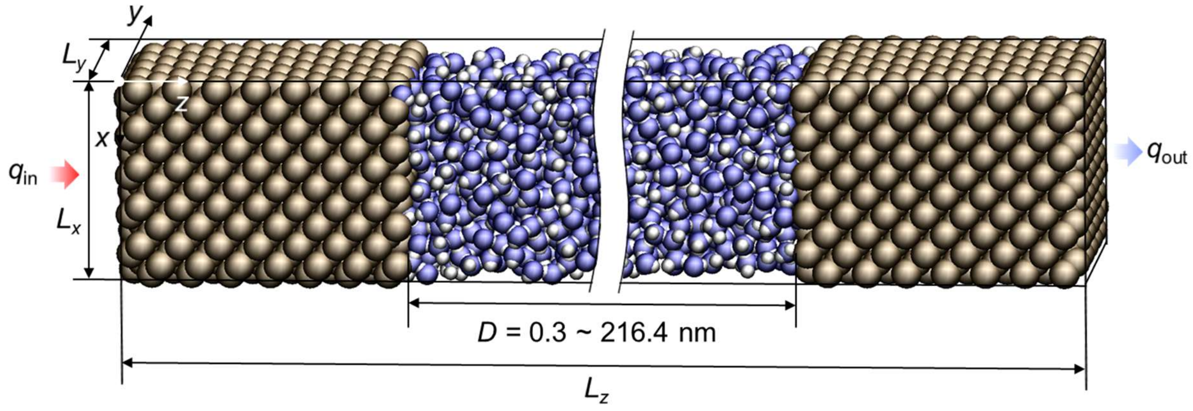

Fig. S12 NEMD simulation system with Si–H_2O interface.

The extended simple point charge (SPC/E) water model [6] was chosen for its effective rigid pair potentials, which include Lennard–Jones (LJ) and Coulombic terms, defined as:

$$\phi(r_{ij}) = 4\varepsilon_{ij}\left[\left(\frac{\sigma_{ij}}{r_{ij}}\right)^{12} - \left(\frac{\sigma_{ij}}{r_{ij}}\right)^{6}\right] + \frac{q_i q_j}{4\pi\varepsilon_0 r_{ij}}, \tag{S2}$$

where r_{ij} is the distance between atoms i and j, ε_{ij} is the depth of the potential well, σ_{ij} is the length parameter, q_i and q_j are the electric charges of atoms i and j, and ε_0 is the vacuum permittivity. The SPC/E model specifies an OH bond length of 1 Å and an H–O–H angle of 109.4°. The SHAKE algorithm was employed to maintain these bond lengths and angles [7]. A cutoff distance of 1 nm was used for the short-range LJ interactions, which affect the oxygen atoms. The particle-particle particle-mesh (PPPM) method, with an accuracy of 10^{-4} was used to calculate long-range Coulombic interactions [8]. Si–Si interactions were computed using the Tersoff potential, while interactions between water molecules and the silicon wall were

modeled through van der Waals forces between silicon and oxygen atoms. The interaction parameters are detailed in Table S9. The silicon–oxygen interactions were calculated using the Lorentz–Berthelot combining rule [9,10].

Table S9 Molecular interaction parameters.

Molecular pair	σ (Å)	ε (eV)	Contact angle (°) [11]	$q_{i,j}$ (e)
O–O	3.166	0.006739	–	–0.8476
H–H	0	0	–	+0.4238
Si–Si	2.095	2.168201	–	0
Si–O	2.6305	0.048352	46.3	–
Si–O	2.6305	0.012088	129.7	–

All simulations were performed using the velocity Verlet algorithm to integrate the equations of motion, employing a time step of 1 fs. Initially, the systems were equilibrated in a canonical ensemble (NVT) at a temperature of 300 K over a period of 5 ns. Subsequently, the thermostat was removed from the liquid, establishing a temperature difference of 20 K. This was achieved by setting the heating and cooling thermostats to 310 K and 290 K, respectively. NEMD simulations were continued for 500 ns to attain steady state in a microcanonical ensemble (NVE). Lastly, data were collected over an additional 25 ns for the determination of temperature and heat flux.

Figure S13 shows the temperature profile in a hydrophilic Si–H_2O simulation system under $\Delta T = 20$ K and $D = 216.4$ nm. The temperatures of both liquid and solid phases correspond well with the linear fit, exhibiting a goodness of fit R^2. The error analysis was performed as shown in Tables S10 and S11. The interfacial temperature remained stable over time, with SE and RSE for l_K below 0.3 nm and 0.2 %, respectively, confirming the high precision of the simulations.

Table S10 Error analysis of temperature gradients in the bulk liquid dT_l/dz, interfacial solid temperature T_{wi}, interfacial liquid temperature T_{li}, interfacial temperature difference ΔT_i, and Kapitza length l_K at the hydrophilic Si–H_2O interface near the heating wall under ΔT = 20 K and D = 216.4 nm.

Time period (ns)	dT_l/dz (K/nm)	T_{wi} (K)	T_{li} (K)	ΔT_i (K)	l_K (nm)
0–5	0.030902	308.136	303.167	4.969	160.799
5–10	0.030907	308.136	303.161	4.975	160.967
10–15	0.030895	308.145	303.167	4.978	161.126
15–20	0.030899	308.137	303.158	4.979	161.138
20–25	0.030904	308.143	303.172	4.971	160.853
average	0.030901	308.139	303.165	4.974	160.977
SE	4.615×10^{-6}	0.004	0.006	0.004	0.155
RSE (%)	0.015	0.001	0.002	0.087	0.096

Table S11 Error analysis of temperature gradients in the bulk liquid dT_l/dz, interfacial solid temperature T_{wi}, interfacial liquid temperature T_{li}, interfacial temperature difference ΔT_i, and Kapitza length l_K at the hydrophilic Si–H_2O interface near the cooling wall under ΔT = 20 K and D = 216.4 nm.

Time period (ns)	dT_l/dz (K/nm)	T_{wi} (K)	T_{li} (K)	ΔT_i (K)	l_K (nm)
0–5	0.030902	291.733	296.690	4.957	160.410
5–10	0.030907	291.734	296.692	4.958	160.417
10–15	0.030895	291.732	296.696	4.964	160.673
15–20	0.030899	291.725	296.692	4.967	160.750
20–25	0.030904	291.721	296.671	4.950	160.173
average	0.030901	291.729	296.688	4.959	160.485
SE	4.615×10^{-6}	0.006	0.010	0.007	0.231
RSE (%)	0.015	0.002	0.003	0.133	0.144

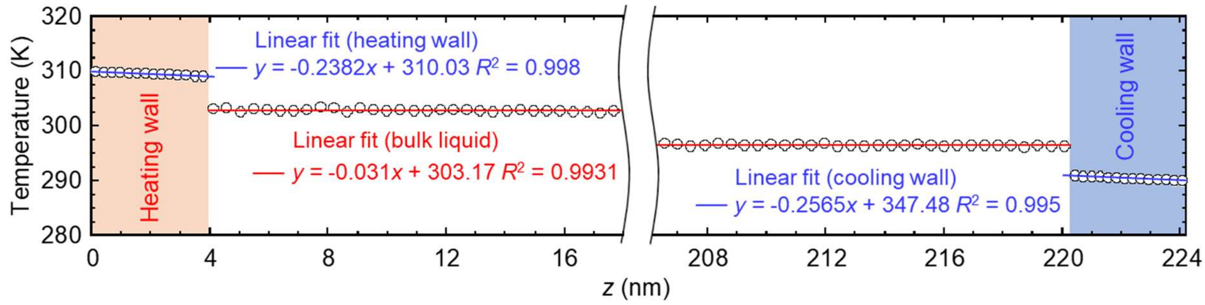

Fig. S13 Temperature profiles for a hydrophilic Si–H_2O simulation system with D = 216.4 nm at ΔT = 20 K.

References

1. Y. Ma, Z. Zhang, J. Chen, K. Sääskilahti, S. Volz, and J. Chen, Carbon N. Y. **135**, 263 (2018).

2. X. Wang, P. Cheng, and X. Quan, Int. Commun. Heat Mass Transf. **77**, 183 (2016).

3. D. Alexeev, J. Chen, J. H. Walther, K. P. Giapis, P. Angelikopoulos, and P. Koumoutsakos, Nano Lett. **15**, 5744 (2015).

4. L. Jin, S. F. M. Noraldeen, L. Zhou, and X. Du, Int. J. Heat Mass Transf. **185**, 122325 (2022).

5. X. Wang and D. Jing, Int. J. Heat Mass Transf. **128**, 199 (2019).

6. H. J. C. Berendsen, J. R. Grigera, and T. P. Straatsma, J. Phys. Chem. **91**, 6269 (1987).

7. S. Miyamoto and P. A. Kollman, J. Comput. Chem. **13**, 952 (1992).

8. S. Plimpton, R. Pollock, and M. Stevens, Proc. Eighth SIAM Conf. Parallel Process. Sci. Comput. 1 (1997).

9. J. Delhommelle and P. Millié, Mol. Phys. **99**, 619 (2001).

10. E. M. Yezdimer, A. A. Chialvo, and P. T. Cummings, J. Phys. Chem. B **105**, 841 (2001).

11. M. Barisik and A. Beskok, Int. J. Therm. Sci. **77**, 47 (2014).
